# Adipose‐derived stem cells enriched with therapeutic mRNA TGF‐β3 and IL‐10 synergistically promote scar‐less wound healing in preclinical models

**DOI:** 10.1002/btm2.10620

**Published:** 2023-11-10

**Authors:** Wei Wang, Liang Chen, Yuxin Zhang, Heng Wang, Dong Dong, Jingjing Zhu, Wei Fu, Tianyi Liu

**Affiliations:** ^1^ Department of Plastic and Aesthetic Surgery Huadong Hospital, Shanghai Medical College, Fudan University Shanghai China; ^2^ Institute of Pediatric Translational Medicine, Shanghai Institute of Pediatric Congenital Heart Disease, Shanghai Children's Medical Center, School of Medicine, Shanghai Jiao Tong University Shanghai China

**Keywords:** adipose‐derived stem cells, ECM remodeling, IL‐10, myofibroblast dedifferentiation, *N* ^1^‐methylpseudouridine, scar formation, TGF‐β3, therapeutic mRNA, wound healing

## Abstract

Skin wound healing often leads to scar formation, presenting physical and psychological challenges for patients. Advancements in messenger RNA (mRNA) modifications offer a potential solution for pulsatile cytokine delivery to create a favorable wound‐healing microenvironment, thereby preventing cutaneous fibrosis. This study aimed to investigate the effectiveness of human adipose‐derived stem cells (hADSCs) enriched with *N* ^1^‐methylpseudouridine (m1ψ) modified transforming growth factor‐β3 (TGF‐β3) and interleukin‐10 (IL‐10) mRNA in promoting scar‐free healing in preclinical models. The results demonstrated that the modified mRNA (modRNA)‐loaded hADSCs efficiently and temporarily secreted TGF‐β3 and IL‐10 proteins. In a dorsal injury model, hADSCs loaded with modRNA TGF‐β3 and IL‐10 exhibited multidimensional therapeutic effects, including improved collagen deposition, extracellular matrix organization, and neovascularization. In vitro experiments confirmed the ability of these cells to markedly inhibit the proliferation and migration of keloid fibroblasts, and reverse the myofibroblast phenotype. Finally, collagen degradation mediated by matrix metalloproteinase upregulation was observed in an ex vivo keloid explant culture model. In conclusion, the synergistic effects of the modRNA TGF‐β3, IL‐10, and hADSCs hold promise for establishing a scar‐free wound‐healing microenvironment, representing a robust foundation for the management of wounds in populations susceptible to scar formation.

**Translational Impact Statement**

This study represents the first investigation into the utilization of mRNA technology in the realm of dermal fibrosis. This innovative platform presents a versatile and secure avenue to foster scar‐less healing by ingeniously integrating classical cytokine and stem cell therapies through the application of modified mRNA technology.

## 1 INTRODUCTION

Skin wound healing relies heavily on tissue fibrosis. While some wounds heal with inconspicuous fibrosis, a considerable proportion of them develop into hypertrophic scars and keloids due to fibroblast hyperplasia and pathological collagen deposition.^1^ The resulting tissue damage can lead to disfigurement, functional limitations, disability, and psychological distress for the patient.^2^, ^3^ The global cost of scar treatment is projected to reach $32 billion by 2027.^4^ Although treatment such as laser therapy, corticosteroid injections, and silicone‐based products can improve scar appearance, complete elimination is challenging.^5^ Surgical excision is often necessary for complete scar removal, and recurrence rates exceed half due to the complex nature of scar formation.^6^ Certain techniques, including creating a moist, tension‐free wound‐healing environment, radiotherapy, and local corticosteroid management, have been employed to reduce the likelihood of scar recurrence, but limited data support their efficacy.^7^ While significant progress has been made in understanding the molecular mechanisms underlying scar formation, no molecular biology therapeutics have yet gained universal acceptance for daily clinical practice.^8^ Given the high incidence of scarring and the lack of effective treatments, there is an urgent need for the development of comprehensive preventative measures following cutaneous injury.

Growth factor and cytokine therapies show promise in improving the wound‐healing microenvironment, but their effectiveness is often hindered by challenges related to stability and delivery methods. Recombinant human interleukin‐10 (IL‐10) and transforming growth factor‐β3 (TGF‐β3) have demonstrated significant efficacy in improving scar formation across multiple animal models.^9^, ^10^ However, clinical trials evaluating these therapies (IL‐10: Prevascar Trial; TGF‐β3: Juvista EU Phase 3 Trial) have not successfully achieved their primary and secondary endpoints.^11^, ^12^ The substantial disparity observed between the effects of exogenous growth factor delivery in preclinical models and clinical trials strongly suggests that relying solely on a single factor is insufficient for achieving scar‐free healing.^13^, ^14^, ^15^, ^16^, ^17^ To better mimic a scar‐free healing microenvironment, an enhanced approach integrating multiple morphogenetic and immunomodulatory factors within a single platform holds the potential to surpass current therapeutic interventions. Recent advancements in transiently expressed modified mRNAs (modRNAs) enable us to further explore this concept.

Various studies have already substantiated that *N* ^1^‐methylpseudouridine‐5′‐triphosphate (m1ψ) modRNA molecules effectively mitigate RNA recognition by innate immune receptors while concurrently enhancing protein expression.^18^, ^19^ They offer a safer gene delivery method compared to DNA/viral‐based technologies, avoid potential allergic and inflammatory risks associated with exogenous protein delivery and improve protein stability.^20^, ^21^ Meanwhile, the combination of chemically modRNAs and cell‐based therapies allows for the design and integration of several therapy modalities on a single delivery platform. These advantages have driven the emergence of messenger RNA (mRNA) applications in regenerative medicine. However, to the best of our knowledge, therapeutic mRNA promoting scar‐less wound healing has not been documented. Classical morphogenetic factor TGF‐β3 and immunosuppressive factor IL‐10 have demonstrated robust effects as recombinant proteins in animal experiments.^22^, ^23^ To further enhance their therapeutic potential, one avenue worth investigating is combining these factors with antifibrotic cellular therapies, aiming to exert synergistic effects.

It is well‐established that adipose‐derived stem cells (ADSCs) exert a multifaceted antifibrotic effect within the wound‐healing microenvironment.^24^ ADSCs possess the remarkable capacity to directly differentiate into crucial cell types and secrete a diverse array of paracrine factors, enabling them to modulate various mechanisms involved in fibrosis. They contribute to extracellular matrix (ECM) degradation, influence the differentiation of fibroblasts into myofibroblasts, positively impact collagen organization, and inhibit the profibrotic effects of TGF‐β1.^25^, ^26^, ^27^ In addition, ADSCs exert influences on elements of both the innate and adaptive immune response systems and stimulate angiogenesis at the injury site through the secretion of proangiogenic cytokines.^28^, ^29^ As a delivery vehicle for mRNA‐based therapeutics, ADSCs exhibit exceptional biocompatibility and safety attributes, stemming from their autologous origin. They effectively mitigate cytotoxicity commonly associated with specific exogenous vectors, and notably, they offer an increased mRNA load capacity.^30^ This capacity has the potential to broaden the range of dosages and extend the effect duration of mRNA therapy.

Here, we use ADSCs as the delivery vehicles for mRNA drugs and their organelles as the tools for mRNA translation and expression to achieve transient, efficient, and pulsatile expression of IL‐10 and TGF‐β3 protein of autologous origin. As a conceptual exploration, the main objective of this study was to evaluate the synergistic effects of modRNA IL‐10‐ and TGF‐β3‐enriched ADSCs (ADSCs^TGF‐β3^, ADSCs^IL‐10^, and ADSCs^dual^) in inhibiting keloid fibroblast (KF) activity, reducing ECM deposition, reversing myofibroblast phenotype, and thus to provide a comprehensive scheme for scar‐susceptible wound management.

## 2 MATERIALS AND METHODS

### 2.1 Cell isolation and culture

Human adipose‐derived stem cells (hADSCs) were isolated from abdominal adipose tissues obtained from liposuction patients. After washing with phosphate‐buffered saline (PBS), the tissues were cut and digested using 0.2% type I collagenase at 37°C for 50 min. A culture medium was then added to halt digestion. For KF isolation, keloid tissues from the abdomen were collected and washed with chloramphenicol solution and PBS, followed by removal of the epidermis. The remaining dermal tissue was minced and subjected to enzyme digestion with 0.3% collagenase II in Dulbecco's modified Eagle medium (DMEM) for 4 h at 37°C. A culture medium composed of DMEM, FBS, and penicillin–streptomycin was used for cell maintenance. Cells were seeded onto culture dishes and cultured for several passages (P3–5). Informed Consent Forms were signed by patients before surgery.

### 2.2 modRNA synthesis and formulation

mRNA was synthesized in vitro using T7 RNA polymerase‐mediated transcription from a linearized DNA template, which incorporates generic 5′‐untranslated region (5′‐UTR) and 3′‐UTR and a poly(A) tail, as previously described.^31^, ^32^ RNA was purified using Ambion MEGAclear spin columns and treated with Antarctic Phosphatase (New England Biolabs) for 30 min at 37°C to remove residual 5′‐phosphates. The RNA was repurified and quantified by Nanodrop (Thermo Fisher Scientific, MA, USA). After purification, modRNA was resuspended in 10 mM Tris HCl and 1 mM EDTA at 1 μg/μL for use. Three different mRNA molecules encoding TGF‐β3 and IL‐10 were modified by the full replacement of uridine‐5‐triphosphate with m1ψ, as previously described.^33^ Open reading frame sequences used for modRNA production were provided in Table S1.

### 2.3 modRNA transfection

The modRNA transfections were executed using MessengerMAX (Invitrogen, Carlsbad, CA, USA) transfection reagents. The in vitro transfections were performed by initially diluting the modRNA and transfection reagents in Opti‐MEM basal media (Invitrogen) and incubating for 5 min. Subsequently, the two solutions were combined and allowed to incubate for an additional 15 min at room temperature (RT) to form RNA–lipid complexes. The transfection of these RNA–lipid complexes was carried out for 4 h, after which the medium was substituted with cell culture media or removed for subsequent cell collection. The transfection reagent was used in a concentration of 2.5 μL MessengerMAX (Invitrogen) per 1 μg RNA, and 1 μg RNA was utilized for the transfection of 100,000 hADSCs.

### 2.4 Construction of hADSCs‐KFs coculture system

To evaluate the impact of modRNA‐transfected ADSCs on KFs, a Transwell coculture system was established. The system utilized Transwell plates with 0.4 μm pores (Corning, NY, USA). ADSCs were seeded in the upper chamber, while KFs were placed in the lower chamber. After both cell types were adhered, modRNAs were introduced into ADSCs via a known transfection protocol. The upper chamber with transfected ADSCs was then moved back to the plate containing KFs. Following a 24‐ to 72‐h incubation period, changes in KF behavior, mRNA expression, and protein expression were assessed.

### 2.5 5‐Ethynyl‐2′‐deoxyuridine proliferation assay

The proliferation of cells was detected using a 5‐ethynyl‐2′‐deoxyuridine (EdU) cell proliferation assay following the manufacturer's instructions. About 1 × 10^5^ cells were seeded in 12‐well plates and incubated for 24 h before the assay. A total of 500 μL EdU (10 μM) reagent (Beyotime, Shanghai, China) was added to each well and incubated for 2 h to label the cells. After three washes with PBS, cells were fixed in a 4% paraformaldehyde solution (R&D System, Minneapolis, MN, USA) for 15 min, permeabilized with 0.3% Triton X‐100 (Beyotime) for an additional 15 min, and then incubated with the click‐reaction reagent for 30 min at RT in a dark environment. Finally, 1× Hoechst 33342 reagent (Thermo Fisher Science) was used to counterstain the nucleus. The stained samples were observed with a fluorescence microscope system, Nikon ECLIPSE Ti‐S, and the data were collected using NIS‐Elements F v4.0 software.

### 2.6 Cell Counting Kit‐8 assay

To assess cell viability, a Cell Counting Kit‐8 (CCK‐8) assay (Beyotime) was conducted following the manufacturer's instructions. Briefly, cells were seeded at a density of 5 × 10^3^ cells per well in a 96‐well plate and allowed to adhere overnight. After treatment with test compounds or vehicle control for 4, 12, 24, 48, and 72 h, the culture medium was removed and replaced with a fresh medium containing 10% CCK‐8 reagent. The cells were incubated at 37°C for 2 h, after which the absorbance at 450 nm was measured using a Thermo Scientific Microplate Reader (SuPerMax 3100). Cell viability was calculated as a percentage of the control cells.

### 2.7 Scratch and transwell migration assay

To study cell migration, a scratch assay was conducted. Cells were seeded in six‐well plates to full confluency. A 10 μL pipette tip was used to create a scratch, followed by medium replacement. Monitoring and imaging occurred at 0, 12, and 24 h using a Nikon ECLIPSE Ti‐S microscope. In addition, migration potential was assessed through a transwell migration assay. Cells were seeded in the upper chamber of a 24‐well Transwell (8 μm pore, corning) with a lower chamber containing 10% fetal bovine serum. After 24 h, nonmigrating cells were removed, and migrating cells were fixed, stained, and observed using the same microscope system, along with NIS‐Elements F v4.0 software for data collection.

### 2.8 Flow cytometry

ADSCs were labeled with fluorescein‐conjugated antibodies (Santa Cruz Biotechnology, CA, USA) targeting CD29, CD44, CD45, CD73, CD90, and HLA‐AR. After harvesting and PBS washing, incubation occurred at 37°C for 30 min in the dark. Following additional washes, cells were resuspended in PBS and analyzed via BD Biosciences flow cytometry. For cell cycle assessment, ADSCs were fixed in 70% ethanol at 4°C for 30 min after PBS wash. Ribonuclease A treatment was used to remove RNA, and propidium iodide (PI; Beyotime) staining was performed. Cell cycle phase distribution (G1, S, G2/M) was determined using flow cytometry along with FlowJo's Cell Cycle Analysis plugin. To analyze apoptosis, ADSCs were exposed to the modRNA‐transfected ADSCs' conditioned medium. After harvesting and washing with PBS, Annexin V‐FITC and PI (Beyotime) were used to label live, early apoptotic, late apoptotic, and necrotic cells. Apoptosis was analyzed using flow cytometry with BD FACSAria SORP and FlowJo software, assessing Annexin V and PI staining.

### 2.9 Quantitative real‐time polymerase chain reaction

Quantitative real‐time polymerase chain reaction (qRT‐PCR) assessed gene expression changes in hADSCs post‐modRNA transfection and KFs upon coculture with modified hADSCs. Total RNA was extracted using an RNA extraction kit (EZBioscience, MN, USA), treated with DNase, and converted into cDNA using a reverse transcription kit (Vazyme, Nanjing, China) from 1 μg RNA. qRT‐PCR was performed using SYBR Green PCR Master Mix (Vazyme) on a Thermo Fisher Scientific real‐time PCR instrument. The cycling conditions included an initial denaturation step (95°C, 10 min), followed by 40 cycles (95°C, 15 s; 60°C, 1 min). Gene expression levels were calculated using the 2^−ΔΔ*C*t^ method and normalized to GAPDH. Primer details are provided in Table S2.

### 2.10 Enzyme‐linked immunosorbent assay

TGF‐β3, IL‐10, MMP1, MMP8, and MMP12 concentrations were measured separately by ELISA Kits (Thermo Fisher Scientific; Cohesion, London, UK; Arigo, Taiwan, China). The absorbance at 450 nm was determined using a 96‐well microplate spectrophotometer (FLUOstar Omega, BMG LABTECH, USA). Four‐parameter logistic curves performed through MyAssays site (https://www.myassays.com/four-parameter-logistic-curve.assay) were used to assay the concentration.

### 2.11 Ex vivo explant culture of keloid tissue

Ex vivo scar tissue was obtained from three patients who had undergone abdominal scar resection. These patients provided informed consent forms after being fully informed. Under aseptic conditions, keloid tissue was harvested, and the epidermis was removed. Keloid fragments were removed from the central region using a 3‐mm circular punch. After being cut to a length of 5 mm, the keloid biopsies were embedded in 24‐well tissue culture plates coated with 0.3 mL growth factor‐reduced Matrigel (BD Biosciences, San Jose, CA, USA). The samples were cultured by the conditioned medium (CM) of hADSCs, ADSCs^TGF‐β3^, ADSCs^IL‐10^, and ADSCs^dual^ separately when the keloid tissue was fixed by the solidified matrix gel. Tissue cultured in a standard medium was used as a control. The culture medium was renewed every 7 days. At 14 days after treatment, the keloid tissue blocks were collected for laboratory tests and analysis.

### 2.12 Western blot

Protein expression analysis involved Western blotting (WB). Cells were lysed using lysis buffer (Epizyme, Shanghai, China), and supernatants were collected postcentrifugation. Protein concentration was determined using a BCA Assay Kit (Thermo Fisher Scientific). Equal amounts of protein (20–50 μg) were separated by SDS‐PAGE (Epizyme) and transferred onto PVDF membranes (Thermo Fisher Scientific). The membranes were blocked with 5% nonfat dry milk in TBST (10 mM Tris HCl, pH 7.4, 150 mM NaCl, 0.05% Tween‐20) for 1 h at RT. Subsequently, the membranes were incubated overnight with primary antibodies, diluted in blocking buffer (Thermo Fisher Scientific). Following TBST washing, the membranes were incubated with HRP‐conjugated secondary antibodies (Beyotime) for 1 h at RT. Membranes were then washed with TBST and developed using an ECL‐Plus Detection Kit (Beyotime). Blots were imaged using a chemiluminescence imager, signal quantification was performed using ImageJ software (National Institutes of Health, Bethesda, MD, USA). Protein levels normalized to β‐tubulin and GAPDH.

### 2.13 Animals and experimental protocol

Male SD rats (8 weeks old, 180‐200 g, SPF) from Beijing Vital River Laboratory Animal Technology Co., Ltd. were used for an in vivo wound‐healing model to evaluate the efficacy of modRNA‐loaded ADSCs in preventing wound‐healing fibrosis. A total of 20 rats were randomly divided into five groups (four rats/group) using a random digit table. Two 1‐cm excisional wounds were created on the back of each rat. A silicone splinting ring was attached with interrupted sutures to prevent premature wound closure. Tegaderm (3M) and self‐adhering elastic bandages covered the wounds. The rats were individually housed in SPF‐grade lab cages. On Days 0, 7, and 14, 0.7 × 10^6^ modRNAs enriched hADSCs in 100 μL PBS were injected around each wound, and 0.3 × 10^6^ cells in 20 μL growth factor‐reduced Matrigel (BD Biosciences) were topically applied. Bandages were removed on Days 5, 10, 15, and 25 for wound observation and photography. The left wound per rat was sampled on Day 18 for histopathological analysis of ECM remodeling. Rats that received a PBS injection were utilized as the control group. Animal procedures were approved by Fudan University's Institutional Animal Care and Use Committee (2023‐HDYY‐102JZS). No specific criteria were established for the inclusion or exclusion of animals, and no data points were excluded during the experiment. To mitigate individual differences and potential interactions between animals, we employed same‐sex rats with closely matched body weights for our experiments. These rats were individually housed in separate cages, all maintained in a uniform rearing environment with consistent dietary conditions. To establish random groupings, we employed the random number table method. Furthermore, to minimize potential bias introduced by experimenter effects, all rats underwent identical experimental procedures, which were consistently administered by a single surgeon. Power analysis using the PWR package in R was used to determine the statistical power of sample size, at the set of statistical significance as 0.05. Collagen volume fraction, evaluated by Masson's staining, served as the primary indicator of power analysis. The power analysis indicated that the sample size of 4 units per group is adequately powered to detect the expected effects, with a statistical power of 1.00.

### 2.14 Morphometric analysis of skin wound area

The skin wound area was photographed and analyzed on Days 0, 5, 10, 15, and 25. The macroscopic wound area was quantified by tracing the wound margin, as determined by photographs taken at various time points. The location of the advancing margin of wound closure was defined as the visibly apparent edge or epithelial migration toward the center of the wound and over the granulation tissue bed. The wound area was calculated as the percentage of the initial wound area $\left(\left[\text{wound area}\;\mathrm{at}\;\text{time}\right]/\left[\text{initial wound area}\right]\times 100\%\right)$ using the ImageJ software.

### 2.15 Hematoxylin and eosin, Masson, picrosirius red staining, immunohistochemistry, immunofluorescence, and confocal microscopy

Picrosirius red, Masson's trichrome, and hematoxylin and eosin (HE) staining for collagen were performed using a Poly Scientific Kit as per the manufacturer's instructions. Images were captured using a Nikon Eclipse TE 300 microscope with Spot Insight CCD camera and MetaMorph software version 6.2 r4. Specimens were fixed in 4% paraformaldehyde, embedded in paraffin, and sectioned at a thickness of 5 μm. Keloid sections were incubated with primary antibodies (1:200 dilution) and visualized with DAB chromogen. Immunofluorescence staining involved blocking with 5% normal goat serum and using primary antibodies diluted in PBS containing 1% goat serum, 0.3% Triton X‐100, and 0.01% sodium azide. Fluorescence signals were detected using a Zeiss LSM710 confocal microscope. Quantitative analysis of various parameters was performed using ImageJ software and its plugins. Polarized images after picrosirius red staining were used for collagen orientation analysis using the Orientation J plugin.

### 2.16 Hyp content assay

Human keloid tissue blocks were homogenized in 2.0 mL distilled water and incubated with 125 μL of 50% trichloroacetic acid on ice for 20 min. Samples were then centrifuged, and the resulting pellets were mixed with 1 mL 12 N hydrochloric acid and baked at 110°C for 14–18 h. The dry samples were dissolved in 200ul of ddH_2_O. Two hundred‐microliter samples or culture fluid (or standards) were added to 0.5 mL 1.4% chloramine‐T in 0.5 M sodium acetate/10% isopropanol (Thermo Fisher Scientific) and incubated for 20 min at RT. Then, 0.5 mL Ehrlich's solution (1.0 M *p*‐dimethylaminobenzaldehyde in 70% isopropanol/30% perchloric acid; Thermo Fisher Scientific) was added, mixed, and incubated at 65°C for 15 min. The optical density of each sample and standard was measured at 550 nm, and the concentration of Hyp was calculated using a four‐parameter logistic curve.

### 2.17 Data processing and statistical analysis

The statistical analyses and visualizations for this study were conducted using R Studio version 4.3.0. Different methods were selected for statistical analysis based on the characteristics of data distribution. One‐way analysis of variance (ANOVA) was used for data that were normally distributed and had homogenous variances. Welch one‐way ANOVA was employed for data that were normally distributed, but had nonhomogeneous variances. For non‐normally distributed data, the Kruskal–Wallis test was utilized. To account for multiple comparisons between two samples, the Bonferroni test was applied. Results were derived from a minimum of three independent experiments, with significance levels denoted as *p < 0.05; **p < 0.01; ***p < 0.001, where *p* < 0.05 indicates statistical significance.

## 3 RESULTS

### 3.1 Synthetic chemically modRNAs are efficiently taken up in ADSCs

**FIGURE 1:**
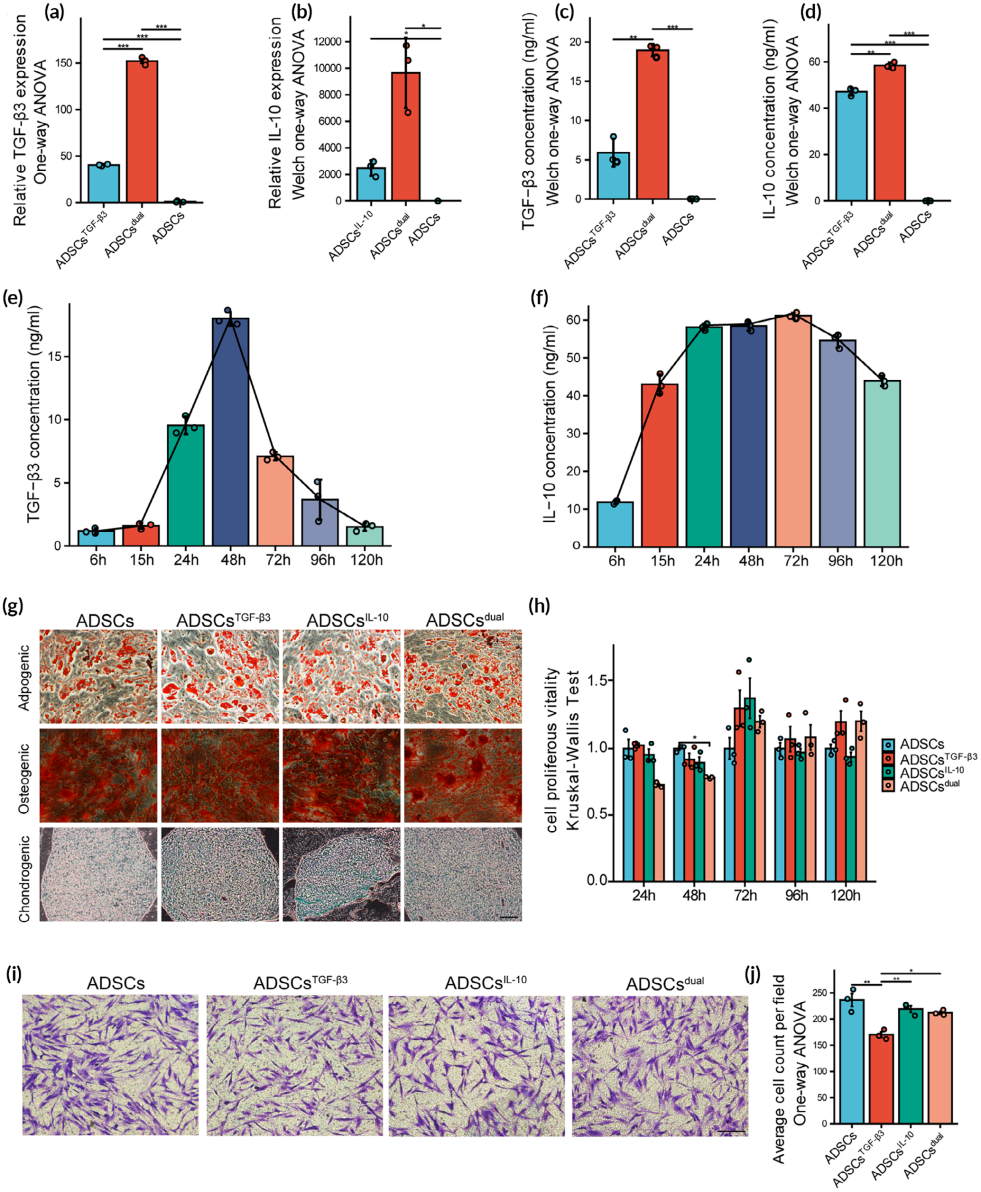
Efficiency of modified mRNA (modRNA) transfection in adipose‐derived stem cells (ADSCs). (A, B) Transcript analysis of intracellular transforming growth factor‐β3 (TGF‐β3) (C) and interleukin‐10 (IL‐10) (D) mRNA levels at 24 h posttransfection. (C, D) Comparison of TGF‐β3 (E) and IL‐10 (F) protein secretion levels at 24 h posttransfection. (E, F) Cumulative protein secretion of TGF‐β3 (G) and IL‐10 (H) in ADSCs^dual^ at different time points (4, 15, 24, 48, 72, 96, and 120 h) posttransfection. (G) Representative images of adipogenic, osteogenic, and chondrogenic differentiation from modRNA‐transfected ADSCs. Successful differentiation of the lineages was stained by Oil Red O, Alizarin Red S, and Alcian blue staining, respectively. Scale bar = 20 μm. (H) Bar graph showing the ADSCs proliferous vitality after 24‐, 48‐, 72‐, 96‐, and 120‐h transfection with modTGF‐β3, modIL‐10, or the both. Cell proliferous vitality was calculated as follows: Cell proliferous vitality = OD (treated) − OD (blank)^mean^/OD (untreated)^mean^ − OD (blank)^mean^. OD represents the optical density value. Untreated primary ADSCs were used as the control. (I) Transwell migration assays on 8‐μm pore size Transwell filters were performed. Representative images of migrated ADSCs after transfection for 24 h were shown. Untreated primary ADSCs were used as the control. (J) Histogram showing the average number of migrated ADSCs per field. Values were presented as mean ± standard deviation (SD), with *n* = 3 samples per group. Statistical significance was determined using *p < 0.05; **p < 0.01; ***p < 0.001.

We initially confirmed the purity of the isolated hADSCs through flow cytometry analysis, which exhibited characteristic surface protein profiles (Figure S1).^34^, ^35^ In our previous studies, we incorporated m1ψ as a substitute for uridine‐5‐triphosphate in the mRNA molecules. These modifications have been demonstrated to enhance mRNA stability and improve protein secretion in various mRNA molecules.^30^, ^36^, ^37^, ^38^ Subsequently, we aimed to assess the efficiency of lipid‐mediated transfection of modRNA molecules encoding TGF‐β3, and IL‐10 (modGFP, modTGF‐β3, and modIL‐10) in hADSCs. Our results revealed that the transfection of modTGF‐β3 and modIL‐10 led to a substantial increase in both gene expression (Figure 1A,B) and protein secretion levels (Figure 1C,D) in hADSCs. Interestingly, co‐transfection of modIL‐10 and modTGF‐β3 exhibited a significantly higher efficiency in terms of gene expression and protein translation compared to individual transfection of modIL‐10 or modTGF‐β3 alone. Furthermore, we accessed the cumulative protein secretion over a 120‐h period following transfection. Our findings indicated that TGF‐β3 concentration peaked at 18 ng/mL after 48 h and decreased by approximately 50% per day (Figure 1E). This decline aligns with the well‐documented high degradation rate of recombinant TGF‐β3 proteins, which can decrease to less than half in 30 min in vitro.^39^ However, the pulsatile delivery via ADSCs maintained a high concentration of TGF‐β3 protein for at least 3 days. In contrast, IL‐10 sustained a high concentration of approximately 55 ng/mg for 96 h after modIL‐10 transfection, and its levels remained stable until the fifth day without a significant decrease (Figure 1F).

We assessed the impact of modTGF‐β3 and modIL‐10 transfections on ADSCs by examining their differentiation, proliferation, and migration capabilities. The findings indicate that the overexpression of mRNAs did not adversely affect the adipogenic, osteogenic, or chondrogenic differentiation capacities of the cells (Figure 1G). The CCK‐8 assay revealed that ADSCs treated with modTGF‐β3 and modIL‐10 exhibited a temporary reduction in proliferative activity within 48 h. Subsequently, their proliferative capacity promptly returned to its original level (Figure 1H). This initial decline in viability could potentially be attributed to liposome‐associated toxicity.^40^, ^41^ In addition, the transwell migration assay showed that ADSCs transfected with IL‐10 exhibited a diminished migratory capability from the moment of transfection up to 24 h thereafter, compared to the other groups (Figure 1I,J).

### 3.2 TGF‐β3 modRNA‐loaded ADSCs inhibit proliferation and migration of KFs

**FIGURE 2:**
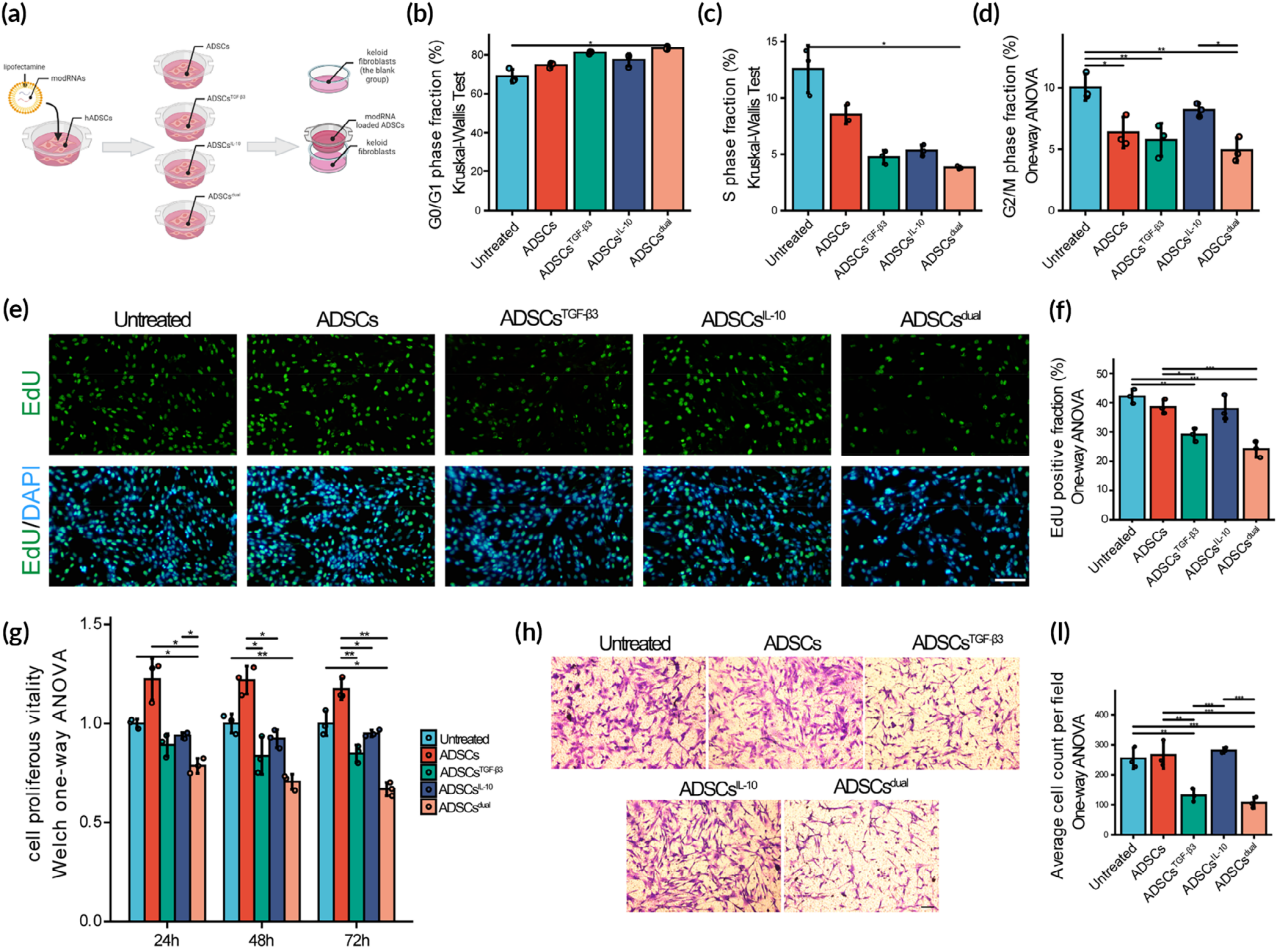
Transforming growth factor‐β3 (TGF‐β3) modified mRNA‐loaded adipose‐derived stem cells (ADSCs) suppressed primary keloid fibroblasts (KFs) proliferation and migration. (A) Schematic representation depicting the coculture procedure. The Transwell coculture system consisted of transfected ADSCs and primary KFs. (B–D) Comparison of cell cycle distribution of KFs cocultured for 24 h, including G0/G1 phase fraction (B), S‐phase fraction (C), G2/M phase fraction (D), and proliferous index (E). Representative fluorescence microscopy images of 5‐ethynyl‐2′‐deoxyuridine (EdU)‐labeled KFs. KFs were cocultured with ADSCs for 24 h. Untreated primary KFs were used as the control (untreated) group. Scale bar = 100 μm. (F) Comparison of the fraction of EdU‐positive KFs among the untreated, ADSCs, ADSCs^TGF‐β3^, ADSCs^IL‐10^, and ADSCs^dual^ groups. (G) Bar graph showing the KFs proliferous vitality after 24, 48, and 72 h treatment with ADSCs, ADSCs^TGF‐β3^, ADSCs^IL‐10^, or ADSCs^dual^. Untreated primary KFs were used as the control. Cell proliferous vitality was calculated as follows: Cell proliferous vitality = OD (treated) − OD (blank)^mean^/OD (untreated)^mean^ − OD (blank)^mean^. OD represents the optical density value. (H) Transwell migration assays on 8‐μm pore size Transwell filters were performed. Representative images of migrated KFs after coculturing with ADSCs, ADSCs^TGF‐β3^, ADSCs^IL‐10^, and ADSCs^dual^ for 24 h were shown. Untreated primary KFs were used as the control. (I) Histogram showing the average number of migrated KFs per field. Values were presented as mean ± standard deviation (SD), with *n* = 3 samples per group. Statistical significance was determined using *p < 0.05; **p < 0.01; ***p < 0.001.

To investigate the regulation of KF viability by hADSCs conditioned with modRNAs, we assessed the cell cycle distribution, apoptosis rate, proliferation rates, and migration capability of KFs after 24 h cocultured with ADSCs, ADSCs^TGF‐β3^, ADSCs^IL‐10^, and ADSCs^dual^. A schematic representation showing the coculture procedure is provided in Figure 2A. The coculture system consisted of transfected or untreated ADSCs in the upper layer and primary KFs in the lower layer. Once the transfection of ADSCs in the upper layer was completed, we replaced the medium with a fresh medium and transferred the ADSCs to a plate containing KFs. After 48 h of coculture, the KFs were used for experiments. Flow cytometry analysis showed that KFs treated with ADSCs^dual^ exhibited a higher proportion of cells in the G0/G1 phase, but a lower proportion in the S and G2/M phases compared to the untreated group, suggesting that modTGF‐β3 conditioned ADSCs significantly inhibit KFs proliferation (Figures 2B,D and S2A–E). The EdU proliferation assay was used to confirm the regulation of KF proliferative activity by ADSCs^TGF‐β3^. The density of EDU‐labeled KFs was significantly lower when cocultured with ADSCs^TGF‐β3^ and ADSCs^dual^ compared to the ADSCs or untreated group, indicating reduced DNA replication (Figure 2E,F). The CCK‐8 assay further confirmed that ADSCs^TGF‐β3^ significantly decreased KF cell viability and proliferation rate, with ADSCs^dual^ demonstrating an even more pronounced effect compared to ADSCs^TGF‐β3^ (Figure 2G). The induction of apoptosis signaling leading to the “self‐destruct” of fibroblasts is considered a crucial step in halting the fibrotic process. Our data suggest that ADSCs alone do not effectively inhibit the apoptosis resistance in fibroblasts. In contrast, KFs cocultured with ADSCs^IL‐10^ and ADSCs^dual^ showed a higher rate of apoptosis following exposure to a water bath at 55°C for 10 min (Figure S2F–K). Scratch assays showed that ADSCs^TGF‐β3^ and ADSCs^dual^‐inhibited KF migration compared to the untreated and ADSCs group (Figure S3). Consistent with the scratch assay, the transwell migration assay showed that the numbers of KFs passing through the microporous membrane after ADSCs^TGF‐β3^ and ADSCs^dual^ treatment were obviously fewer than the untreated and ADSCs group (Figure 2H,I).

### 3.3 modRNA‐loaded ADSCs upregulate MMP expression in vitro

**FIGURE 3:**
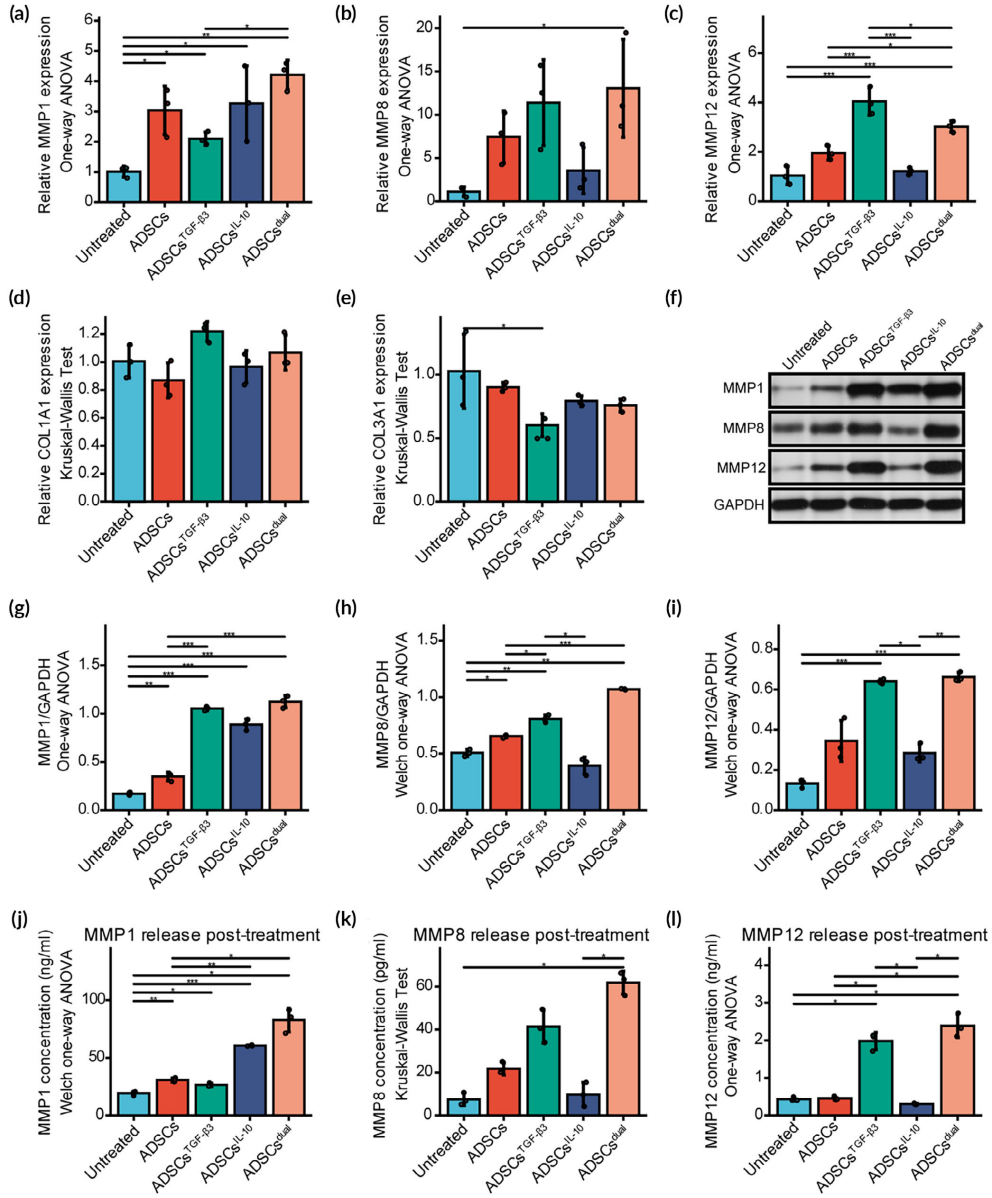
Adipose‐derived stem cells (ADSCs) enriched with transforming growth factor‐β3 (TGF‐β3) and interleukin‐10 (IL‐10) modified mRNA significantly enhanced MMP1, MMP8, and MMP12 secretion. (A–E) Transcript analysis of extracellular matrix (ECM)‐related genes, including MMP1 (A), MMP8 (B), MMP12 (C), COL1A1 (D), and COL3A1 (E) in keloid fibroblasts (KFs) cocultured with ADSCs, ADSCs^TGF‐β3^, ADSCs^IL‐10^, and ADSCs^dual^ after 48 h. Untreated primary KFs were used as the control. (F) Western blot analysis of intracellular ECM‐related genes, including MMP1, MMP8, and MMP12, from KFs cocultured for 48 h. Full‐length blots/gels are presented in Figure S6. (G–I) Comparison of the ECM‐related protein expression, including MMP1 (G), MMP8 (H), and MMP12 (I). GAPDH served as a loading control. (J–L) After treating ADSCs, ADSCs^TGF‐β3^, ADSCs^IL‐10^, and ADSCs^dual^ for 48 h, ADSCs were removed, and serum‐free medium was replaced to continue incubating KFs for 24 h. MMP1 (J), MMP8 (K), and MMP12 (L) secretion of KFs were determined by enzyme‐linked immunosorbent assay. Untreated primary KFs were used as the control. Values were presented as mean ± standard deviation (SD), with *n* = 3 samples per group. Statistical significance was determined using *p < 0.05; **p < 0.01; ***p < 0.001.

To investigate the impact of modRNAs delivered by ADSCs on ECM remodeling and turnover, we conducted qRT‐PCR analysis of ECM‐related genes in KFs cocultured with transfected ADSCs for 48 h (Figure 3A–E). Significant alterations in the expression levels of matrix metalloproteinases (MMPs) were observed. ADSCs^TGF‐β3^ treatment upregulated MMP1 and MMP12 compared to the untreated groups. ADSCs^dual^ upregulated MMP12 expression in KFs compared to the untreated and ADSCs groups, and also showed overexpression of MMP1 and MMP8 compared to the untreated group. However, no statistically significant differences were observed in the expression of collagen‐encoding genes COL1A1 and COL3A1, except for the downregulation of COL3A1 by ADSCs^TGF‐β3^, indicating a limited effect on collagen synthesis.

WB analysis was performed (Figure 3F) to validate the impact of modRNA‐loaded ADSCs on protein expression levels. Notably, MMP1 and MMP8 were upregulated in the ADSCs^TGF‐β3^ and ADSCs^dual^ groups compared to the untreated and ADSCs groups (Figure 3G,H). MMP12 expression was significantly increased in the ADSCs^TGF‐β3^ and ADSCs^dual^ groups compared to the untreated group (Figure 3I). Meanwhile, ADSCs^IL‐10^ treatment resulted in a significant upregulation of MMP1 expression compared to KFs treated with ADSCs alone or untreated control KFs.

Subsequently, we aimed to investigate the potential of modRNA‐loaded ADSCs therapy in regulating MMP secretion, thereby further validating our previous findings. To assess the impact of transfected ADSCs on MMP secretion in KFs, we cocultured them for 48 h. Subsequently, we removed the ADSCs and cultured the KFs in a serum‐reduced medium for an additional 24 h. Following this, we measured the concentration of secreted MMP1, MMP8, and MMP12 by the KFs (Figure 3J–L). The enzyme‐linked immunosorbent assay (ELISA) results demonstrated that ADSCs^dual^ significantly increased the secretion of MMP1, MMP8, and MMP12. In addition, the ADSCs^IL‐10^ group exhibited notably higher levels of MMP1 secretion, while the ADSCs^TGF‐β3^ group showed elevated levels of MMP8 and MMP12 secretion.

### 3.4 IL‐10 modRNA‐loaded ADSCs facilitate myofibroblast dedifferentiation

**FIGURE 4:**
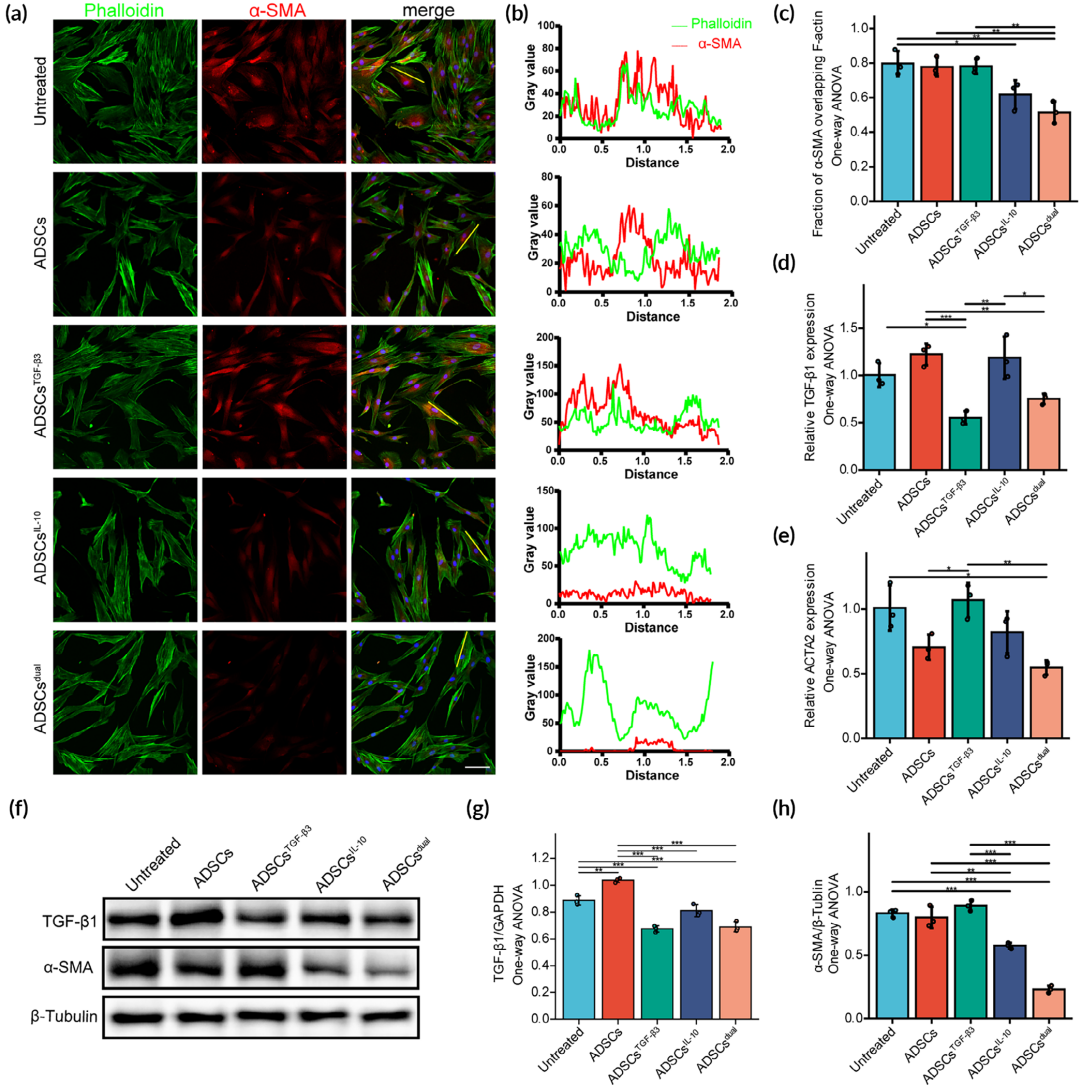
Adipose‐derived stem cells (ADSCs) enriched with interleukin‐10 (IL‐10) modified mRNA reversed myofibroblast phenotype in vitro. (A) Immunofluorescence staining of α‐smooth muscle actin (α‐SMA; shown in red) in keloid fibroblasts (KFs) exposed to ADSCs, ADSCs^TGF‐β3^, ADSCs^IL‐10^, and ADSCs^dual^. Phalloidin (shown in green) and DAPI (shown in blue) were used for co‐staining. Primary KFs were used as a control. Scale bar = 100 μm. (B) Waveform plots on the right side of the fluorescence microscopy images reflect the fluorescence intensity of stress fibers (green) and α‐SMA (red) at the locations marked by yellow lines. (C) Comparison of the fraction of α‐SMA (red) overlapping with F‐actin (green) using the Colocalization Finder plugin of ImageJ. F‐actin was marked by phalloidin (green). (D, E) Comparison of the relative expression level of transforming growth factor‐β1 (TGF‐β1) (D) and ACTA2 (E) mRNA among KFs cocultured with ADSCs, ADSCs^TGF‐β3^, ADSCs^IL‐10^, and ADSCs^dual^ for 48 h. Primary KFs were used as a control. (F) Western blot analysis of TGF‐β1 and α‐SMA expression in KFs treated for 48 h. Primary KFs were used as a control. Full‐length blots/gels are presented in Figure S7. (G, H) Quantification of TGF‐β1 and α‐SMA expression by grayscale value analysis. β‐Tubulin served as a loading control. Values were presented as mean ± standard deviation (SD), with *n* = 3 samples per group. Statistical significance was determined using *p < 0.05; **p < 0.01; ***p < 0.001.

Previous studies have shown that ADSCs can reduce ACTA2 transcription levels in vitro, and IL‐10 has been found to reverse TGF‐β1‐induced myofibroblast differentiation.^25^, ^42^, ^43^, ^44^ To investigate the potential of IL‐10 delivery via ADSCs to promote myofibroblast regression in vitro, we evaluated the changes in α‐smooth muscle actin (α‐SMA) and TGF‐β1 expression in human keloid‐derived fibroblasts. High expression of α‐SMA in stress fibers is a major feature of fibroblast activation into myofibroblasts (contractile phenotype). Images of immunofluorescence revealed untreated control KFs appeared to assemble mature α‐SMA‐containing stress fiber filaments, while the α‐SMA expression significantly reduced in stress fibers when treated with ADSCs, ADSCs^IL‐10^, and ADSCs^dual^. Representative images are shown in Figure 4A,B. Furthermore, co‐localization analysis revealed a reduced fraction of α‐SMA overlapping with F‐actin (Figure 4C). The results of transcript analysis suggested that the relative expression of TGF‐β1 and ACTA2 was significantly reduced in KFs treated with ADSCs^dual^ (Figure 4D,E). Immunoprotein blotting for TGF‐β1 and α‐SMA revealed that untreated primary KFs normally expressed intense TGF‐β1 and α‐SMA. The expression of α‐SMA was significantly decreased in KFs treated with ADSCs^IL‐10^ and ADSCs^dual^, while the TGF‐β1 expression was downregulated in KFs treated with ADSCs^TGF‐β3^ and ADSCs^dual^ (Figure 4F–H).

### 3.5 modRNA‐loaded ADSCs reduce cutaneous fibrosis during wound healing in rat

**FIGURE 5:**
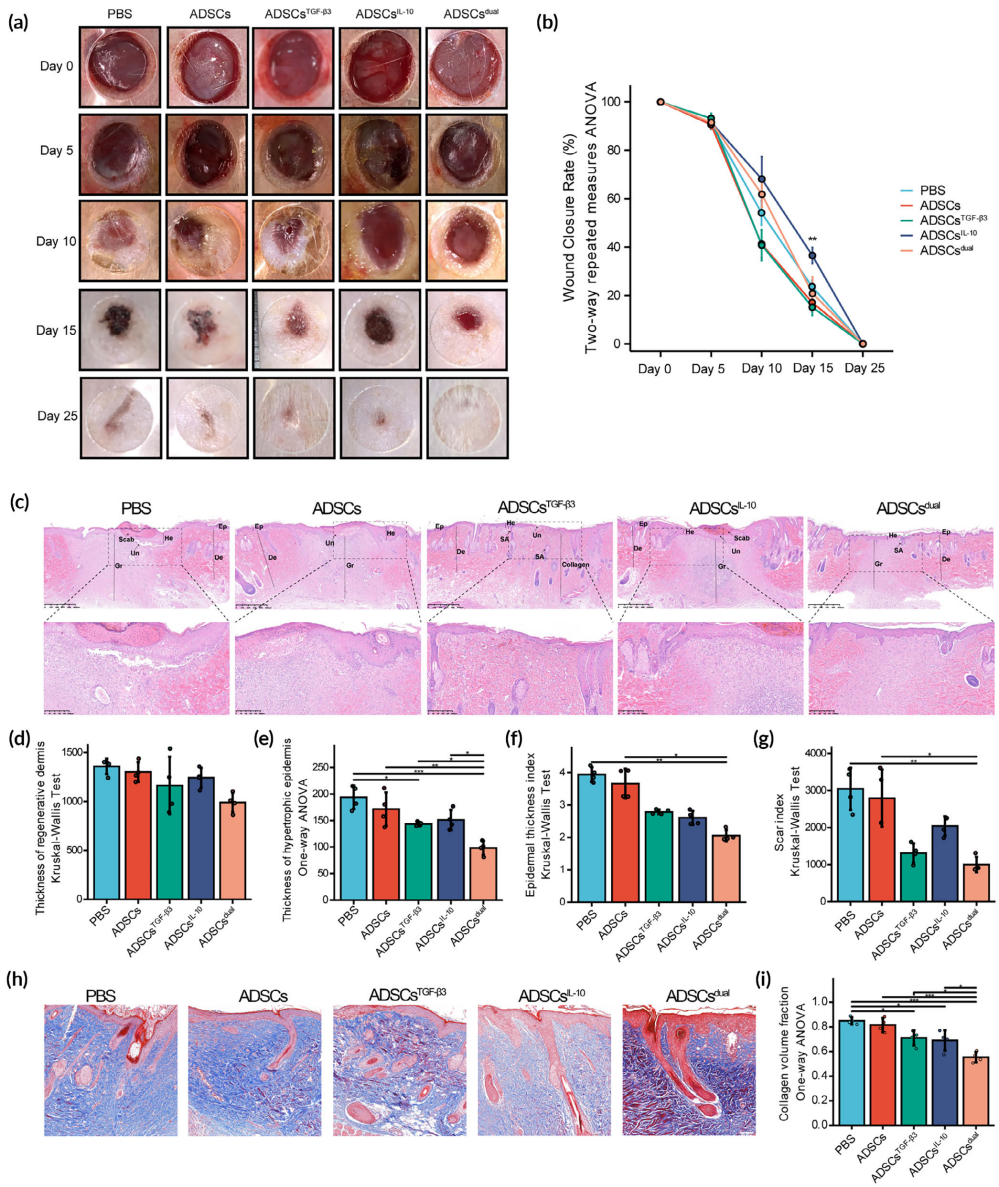
Transforming growth factor‐β3 (TGF‐β3) and interleukin‐10 (IL‐10) loaded adipose‐derived stem cells (ADSCs) significantly attenuated cutaneous fibrosis in rat dorsal injury. (A) Representative photographs of the wound area on postoperative days (PODs) 0, 5, 10, 15, and 25, showing the progress of wound closure and scar formation after injury. Wound beds were administrated with ADSCs, ADSCs^TGF‐β3^, ADSCs^IL‐10^, or ADSCs^dual^ as treatment. Rats injected with phosphate‐buffered saline (PBS) served as the control. (B) Quantification of wound closure rate on PODs 5, 10, 15, and 25. The wound closure rate was calculated as the percentage of wound closure compared to the initial wound size. (C) Representative images showing hematoxylin and eosin (HE) staining of wound bed harvested on POD 20 in each group. The scab, unwound area (Un), hyperplastic epidermis (He), normal epidermis (Ep), granulation tissue (Gr), dermis (De), skin appendages (SA), and collagen tissue (collagen) were labeled in the corresponding site. Scale bar = 625 μm. (D–G) Comparison of parameters for quantifying scar formation between each group. Rats injected with PBS served as the control. Depth of fibrous tissue (D), measured as the distance from the surface of the wound bed to the deepest point of fibrosis. Thickness of hypertrophic epidermis (E), measured as the distance from the top of the hyperplastic epidermis to the top of the dermis underneath it. Epidermis thickness index (F), calculated as the ratio of the thickness of the hypertrophic epidermis to the thickness of the normal epidermis. Scar index (G), calculated as the ratio of the area of fibrous tissue to the normal dermis depth. (H) Representative images showing Masson's trichrome stained regenerative tissue on POD 20, showing the collagen fibers (blue) and muscle fibers (red) in the tissue. Scale bar = 100 μm. (I) Comparison of total collagen volume fraction (defined as area fraction of collagen fibers) from Masson's trichrome staining between the modRNA‐loaded ADSCs group and the PBS (control) group. Values were shown as mean ± standard deviation (SD), with *n* = 4 rats per group. Statistical significance was determined using *p < 0.05; **p < 0.01; ***p < 0.001.

To investigate the potential of ADSCs delivering modRNAs in preventing skin fibrosis, we employed it in dorsal wound models in rats, which closely mirror the biological processes of human wound healing. A schematic of the animal experimental approach is shown in Figure S4A. In the initial stages, wounds subjected to different treatments did not show significant differences in the rate of healing. However, wounds treated with ADSCs^IL‐10^ demonstrated a lower rate of wound closure compared to the control group on postoperative day (POD) 15. Nevertheless, by POD 25, all rat wounds had completely closed. Wounds treated with ADSCs^IL‐10^, ADSCs^TGF‐β3^, and ADSCs^dual^ showed reduced elevation, depression, and erythema compared to the control group and wounds treated with ADSCs alone (Figure 5A,B and S4B).

Histological analysis was conducted to assess the wound bed treated with modRNA‐conditioned ADSCs after 18 days of administration. Initially, HE staining was employed to visualize the regenerated skin structure at the wound site and quantify indexes such as hypertrophic dermis, granulation or fibrotic tissue, scar formation, and epidermal thickness.^45^ In HE staining images (Figure 5C), the PBS group showed an immature scab and the presence of granulation tissue with a high level of inflammatory cell infiltration underneath the discontinuous and hypertrophic epidermal layer. In addition, the fibrosis formed around the granulation tissue was thicker and had no regenerated skin appendages. Compared to the PBS group, the ADSCs‐treated group showed smaller unclosed wounds, but no significant improvement in other indicators. The ADSCs^TGF‐β3^‐ and ADSCs^dual^‐treated wound had thinner epidermal thickness, lower scar index and epidermal thickness index, and exhibited well‐integrated regenerative skin appendages in the immatured fibrotic healed tissue, indicating improved reconstruction of epithelial and dermal layers (Figure 5D‐G). In the ADSCs^IL‐10^‐treated group, the area of unclosed wounds and granulation tissue was more extensive compared to the other groups, and there was a higher level of inflammatory cell infiltration on POD 18, indicating delayed wound healing. Masson staining was performed to assess the content and distribution of regenerative collagen in the remodeling area beneath the hypertrophic epidermal layer on POD 18 (Figure 5H). As anticipated, the wounds treated with ADSCs^TGF‐β3^ and ADSCs^IL‐10^ exhibited significantly lower collagen density compared to the PBS treatment group. Furthermore, the wounds treated with ADSCs^dual^ showed even lower collagen density compared to the PBS group and the ADSCs‐treated group (Figure 5I).

### 3.6 ADSCs^dual^ improves collagen organization, vascularization, and promotes myofibroblast clearance during the tissue remodeling phase

**FIGURE 6:**
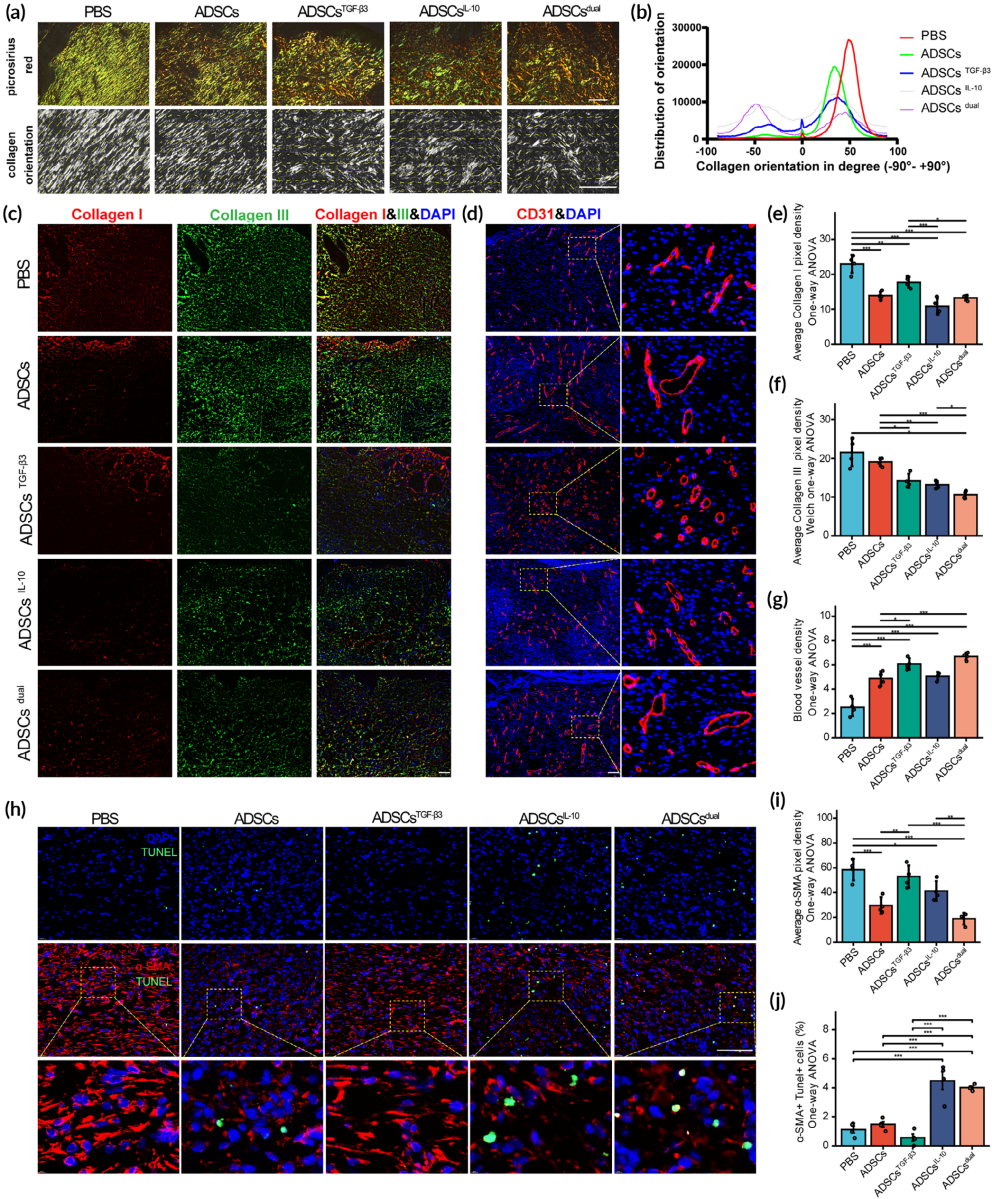
Transforming growth factor‐β3 (TGF‐β3) and interleukin‐10 (IL‐10) modified mRNA‐loaded adipose‐derived stem cells (ADSCs) improved collagen organization, collagen deposition, and promoted neovascularization in regenerative area. (A) Representative images showing picrosirius red staining under polarized light (upper). Vector field images of picrosirius red staining (bottom) showing collagen alignment direction in regenerative dermis harvested on postoperative day (POD) 20. Scale bar = 100 μm. (B) Waveform graph depicting the distribution of collagen fibers in different orientations. (C) Representative images of immunofluorescence staining in the regenerative dermis for collagen I (red) and collagen III (green) exposed to ADSCs, ADSCs^TGF‐β3^, ADSCs^IL‐10^, and ADSCs^dual^. The phosphate‐buffered saline‐treated rats served as the control. DAPI (blue) was used for nuclear staining. Samples were harvested on POD 20. (D) Representative images of immunofluorescence staining for CD31 (red) in granulation tissue to label the neovascularization on POD 20. Scale bar = 100 μm. (E, F) Comparison of average pixel fluorescence density of collagen I (E) and collagen III (F) in regenerative dermis, using average pixel density calculated by ImageJ. (G) Comparison of blood vessel density using the VesselJ plugin of ImageJ. (H) Representative images of immunofluorescence staining of α‐smooth muscle actin (α‐SMA) (red) in tissue remodeling areas of regenerative dermis on POD 20, co‐stained with terminal deoxynucleotidyl transferase dUTP nick end labeling (TUNEL) (green) and DAPI (blue). (I, J) Comparison of average pixel fluorescence density of α‐SMA (I) and α‐SMA positive TUNEL positive cell rates (J) in the tissue remodeling regions. Values were shown as mean ± standard deviation (SD), with *n* = 4 rats per group. Statistical significance was determined using *p < 0.05; **p < 0.01; ***p < 0.001.

To assess collagen organization (i.e., the alignment of collagen fibers), which is closely related to scar formation, we conducted the picrosirius red stain assay. Under polarized light (Figure 6A), the untreated group showed denser collagen bundles with more fine type III collagen (shows green color in polarized light) compared to other groups. This tendency was also observed in the ADSCs‐treated group, albeit with much sparser bundles than the untreated group. In contrast, ADSCs^TGF‐β3^‐, ADSCs^IL‐10^‐, and ADSCs^dual^‐treated groups displayed thicker woven networks of collagen bundles, with collagen type III bundles being gradually replaced by collagen type I bundles. In addition, the ADSCs^IL‐10^‐, ADSCs^TGF‐β3^‐, and ADSCs^dual^‐treated groups exhibited mostly randomly distributed collagen bundles, while the untreated and ADSCs‐treated groups show a directional distribution of collagen with higher brightness (Figure 6B). Collectively, the modRNA‐loaded ADSCs groups showed greater distribution, resembling a basket‐weave formation, which is more similar to the collagen distribution seen in normal dermis. In immunofluorescence staining analysis of regenerative tissue, the ADSCs^dual^‐treated group showed the lowest collagen density, followed by ADSCs^IL‐10^‐ and ADSCs^TGF‐β3^‐treated groups (Figure 6C). Collagen I staining was significantly reduced in wounds treated by ADSCs, ADSCs^TGF‐β3^, ADSCs^IL‐10^, and ADSCs^dual^, while collagen III deposition was only regulated by ADSCs^dual^ (Figure 6E,F). Active neovascularization plays a crucial role in promoting wound healing by facilitating the provision of an adequate blood supply and balancing the inflammatory response, thereby reducing the risk of excessive scar formation.^46^ We qualified the neovascularization in regenerative tissues through CD31 staining (Figure 6D). As shown in Figure 6G, ADSCs treatment remarkably increased the density of blood vessels in the regenerative tissue, and this effect was further amplified when ADSCs were combined with TGF‐β3 (ADSCs^TGF‐β3^) or administered in a dual approach (ADSCs^dual^). Although some publications have suggested that ACTA2 expression is merely an in vitro phenomenon and may not be present in the dermis with pathologic scars,^47^ the concept of promoting myofibroblast clearance through dedifferentiation and apoptosis during the wound remodeling stage (occurring after 18–21 days postinjury) has still emerged as a potential strategy for effectively mitigating excessive fibrosis and scarring.^48^ Inspired by the contentious nature of current research and supported by prior in vitro observations, which demonstrated that the application of ADSCs^IL‐10^ and ADSCs^dual^ results in a noteworthy decrease in apoptosis resistance and a reversal of the myofibroblast phenotype in primary KFs, our study sought to assess the impact of ADSCs^dual^ on myofibroblasts within the tissue remodeling zone in an in vivo setting. We conducted immunofluorescence labeling to assess α‐SMA expression and employed the terminal deoxynucleotidyl transferase dUTP nick end labeling (TUNEL) method to identify apoptotic cells within the tissue remodeling area underlying the thickened epidermis (Figure 6H). Dorsal injury induced an intense α‐SMA expression in the control condition, while the expression was significantly reduced in wound‐healing areas treated with ADSCs, ADSCs^IL‐10^, and ADSCs^dual^ (Figure 6I). The quantitative analysis of α‐SMA positive TUNEL positive cells revealed a markedly higher rate of α‐SMA positive cell apoptosis in ADSCs^IL‐10^‐ and ADSCs^dual^‐treated wounds, as compared to both the PBS, ADSCs‐ and ADSCs^TGF‐β3^‐treated groups (Figure 6J).

### 3.7 ADSCs^TGF‐β3^ and ADSCs^dual^ induce collagen degradation ex vivo

**FIGURE 7:**
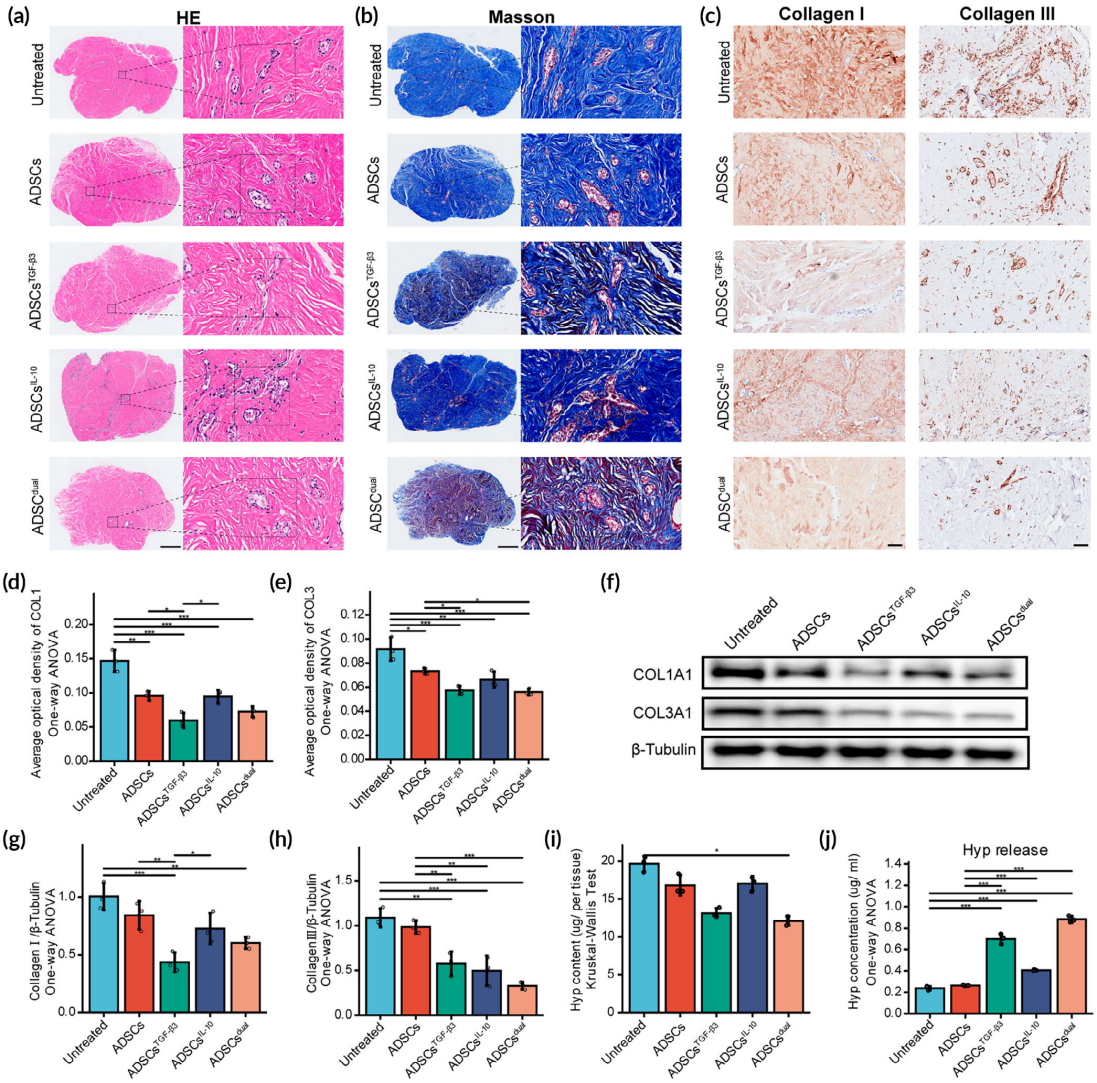
ADSCs^TGF‐β3^ and ADSCs^dual^ promote extracellular matrix degradation in the keloid explant model. (A) Keloid explants were treated with a conditioned medium generated by ADSCs, ADSCs^TGF‐β3^, ADSCs^IL‐10^, and ADSCs^dual^ for 14 days. Representative images of hematoxylin and eosin staining showing the cellular and fiber structure in keloid explants. Scale bar = 500 μm. (B) Representative images of Masson's trichrome staining reflecting the collagen content of keloid explants. Scale bar = 500 μm. (C) Representative images of immunohistochemistry for collagen I and collagen III. Scale bar = 100 μm. (D, E) Comparison of the average optical density of collagen I (D) and collagen III (E). (F) Western blot analysis of collagen I and III in cultured keloid explants. Full‐length blots/gels are presented in Figure S8. (G, H) Quantitation of collagen I and III expression by grayscale value analysis. β‐Tubulin was used as a reference gene. (I) Histograms showing the content of hydroxyproline (Hyp) in keloid explants. (J) Histograms showing the content of hydroxyproline (Hyp) in culture medium, representing the Hyp released from the keloids. Values are presented as mean ± SD, *n* = 3/group, *p < 0.05; **p < 0.01; ***p < 0.001. ADSCs, adipose‐derived stem cells; IL‐10, interleukin‐10; TGF‐β3, transforming growth factor‐β3.

Our previous data have demonstrated the efficacy of ADSCs loaded with modRNA in suppressing the activity of primary KFs and significantly reducing transient scar formation in animal wound models. To further substantiate their potential for clinical application, we employed a keloid explant model to meticulously evaluate their efficiency in attenuating collagen disposition. The schematic of the ex vivo explant culture of keloid tissue is shown in Figure S5. After 14 days of administration, the CM derived from ADSCs^TGF‐β3^ and ADSCs^dual^ exhibited significant reductions in cellularity, microvasculature, and ECM content within keloid tissues (Figure 7A,B). Immunoreactivity of type I and type III collagen was significantly diminished in keloids treated with ADSCs^TGF‐β3^‐CM and ADSCs^dual^‐CM, in comparison to the other groups (Figure 7C–E). To further confirm these findings, we extracted type I and type III collagen from keloid explants and determined their expression by WB (Figure 7F–H). In line with the immunohistochemical results, the expression of type I and type III collagen was significantly lower in the ADSCs^TGF‐β3^‐CM‐ and ADSCs^dual^‐CM‐treated keloid tissue compared to the untreated and ADSCs groups. In addition, the total hydroxyproline content, a well‐established indicator of total collagen content, was significantly decreased in keloid explants treated with ADSCs^TGF‐β3^‐CM and ADSCs^dual^‐CM (Figure 7I). Of note, a higher concentration of hydroxyproline was released into the culture in ADSCs^TGF‐β3^‐CM‐ and ADSCs^dual^‐CM‐treated keloids (Figure 7J), implying a higher collagen metabolizing activity.

## 4 DISCUSSION

Progress in wound healing has been achieved using ADSCs, but enhancing the clinical applicability requires careful consideration. ADSCs are sometimes genetically modified based on DNA/viral techniques to enhance their specific functions, tailored to the characteristics of the disease at hand.^49^ However, it is important to address the potential concerns related to adverse effects and safety before considering the clinical translation of this approach. In recent studies, our team has documented the advantageous outcomes of a cell‐based mRNA delivery platform for enhancing therapeutic applications in limb ischemia, myocardial infarction, bone defects, corneal injury, and improvement of fat grafting. These studies have yielded promising results.^30^, ^36^, ^37^, ^38^, ^50^ As an exploration in the field of scar‐less healing, we developed ADSCs^dual^, which can continuously express IL‐10 and TGF‐β3 using liposomal transient transfection of modRNA IL‐10 and TGF‐β3.

While recombinant human TGF‐β3 and IL‐10 have shown promise in improving or preventing scarring in preclinical studies, clinical trials involving TGF‐β3 (Juvista EU Trial) and IL‐10 (Prevascar Trial) were halted due to inadequate efficacy endpoints. The effectiveness of growth factors is limited by factors such as low stability, a short in vivo half‐life, and the potential for adverse effects stemming from elevated local and/or systemic concentrations. The unexpected unfavorable outcomes of the TGF‐β3 trial have been attributed to the use of a lower dosage in the phase III trial compared to the amount tested in previous phase I/II trials.^51^ Furthermore, there appears to be a recurring trend where interventions targeting specific aspects or factors of fibrotic conditions often fail to yield significant clinical benefits.^52^ Autologous ADSCs, when equipped with therapeutic mRNAs, hold the potential to produce autologous‐derived TGF‐β3 and IL‐10 in a pulsatile manner. This innovative approach offers a new avenue for addressing the issues stemming from their brief in vivo half‐life and single‐mode action. Herein, we present compelling evidence for the immense therapeutic potential of modTGF‐β3‐ and modIL‐10‐enriched ADSCs in promoting ECM metabolism, myofibroblast regression, and angiogenesis based on in vivo, in vitro, and ex vivo preclinical models.

The coadministration of TGF‐β3 and IL‐10 have already been investigated by Park et al. They developed a coacervates‐mediated TGF‐β3 and IL‐10 delivery platform that could enhance the activity of normal dermal fibroblast, regulate the expression of scar‐related genes, and promote scar‐less regeneration.^53^ However, ADSCs‐delivered modRNAs possess several advantages compared to exogenous delivery platforms that rely on recombinant protein technology and biocompatible carriers. First, utilizing autologous ADSCs allows for the endogenous secretion of proteins, reducing concerns about allergic reactions associated with exogenous proteins and materials. Second, ADSCs exert their synergistic therapeutic effects through their potent paracrine action and extracellular vesicular transport, facilitating the delivery of a diverse repertoire of noncoding RNAs, growth factors, and cytokines. The use of different combinations of modified RNAs to induce enhanced expression of specific cytokines and growth factors provides greater flexibility in treatment options. In addition, while Park et al.'s study provided limited experimental evidence of the synergistic efficiency of the dual GF administration, especially in the reversal of specific pathogenesis, this current study has made considerable efforts to investigate the impact of different treatments (ADSCs/ADSCs^IL‐10^/ADSCs^TGF‐β3^/ADSCs^dual^) on the biological behavior of primary KFs, rat skin fibrosis, and in vitro cultured keloid tissue. The synergistic effect between the three is promising. Compared with ADSCs or ADSCs loaded with IL10 or TGF‐β3 alone, ADSCs^dual^ more significantly increased the expression of MMPs and inhibited myofibroblast activation and collagen deposition levels. It successfully counteracted the delayed wound healing caused by ADSCs^IL‐10^. Most notably, we observed remarkable regeneration of skin appendages in both wounds treated with ADSCs^TGF‐β3^ and ADSCs^dual^, suggesting that our treatment protocols can achieve a repair effect that closely resembles that of normal dermis.

An important finding was the substantial increase in both the expression and secretion of matrix metalloproteinases in KFs following treatment with modRNA‐loaded ADSCs. Among the various MMPs we identified for upregulation, MMP1 and MMP8 are the primary collagenases responsible for breaking down various types of interstitial collagen, while MMP12 is an elastase primarily involved in the degradation of elastin, which plays a crucial role in tissue remodeling and repair.^54^ While MMPs play a beneficial role in facilitating the degradation of the ECM and consequently impeding fibrosis,^55^ a considerable number of studies provided evidence of the correlation between elevated MMPs and delayed wound healing.^56^ Hence, in our pursuit of a balanced approach to the impact of elevated MMPs on wound‐healing and skin fibrosis, we must consider several key factors. First, precision treatment is essential. Identifying suitable patients, where the benefits outweigh potential risks like delayed wound healing due to MMP overexpression, remains a challenge. Developing risk‐scoring systems based on clinical and genetic markers could aid in patient selection. Second, it is crucial to clarify the role of MMP levels in the wound‐healing process. While excessive MMP expression is observed in chronic wounds with delayed healing, MMPs are also involved in essential physiological processes like inflammation, angiogenesis, re‐epithelialization, and ECM remodeling during normal wound healing. If we can identify the best marker threshold to predict the clinical evolution of the pathology, it may be possible to regulate MMPs at distinct locations and times. Finally, while our study did not focus on administration time windows and dosages, they are crucial for future clinical applications. Larger, comprehensive studies are needed to address safety concerns related to clinical translation.

In this study, another important discovery is that treatment with ADSCs^IL‐10^ or ADSCs^dual^ significantly reversed the myofibroblast phenotype in vivo. Recent advances have revealed that high levels of IL‐10 can facilitate full dedifferentiation of myofibroblasts back into fibroblasts in a three‐dimensional culture model.^43^, ^44^, ^57^ In the context of immunofluorescence images depicting the wound remodeling phase, our observations indicate that the use of IL‐10‐loaded ADSCs as a treatment modality leads to a notable increase in apoptosis along with a noticeable reduction in α‐SMA expression within the subset of α‐SMA positive cells. Contrary to some previous suggestions that enhanced α‐SMA expression in fibroblasts is mainly an in vitro phenomenon and almost absent in mature scar tissue, our findings emphasize the dynamic shifts in α‐SMA expression within the in vivo wound‐healing microenvironment as pivotal regulatory focal points in dermal fibrosis. Considering the intricate nature of the in vivo microenvironment, our data offer a novel reference for further research in this field. On the other hand, delayed wound healing was observed in ADSCs^IL‐10^‐intervened rats on POD 15, although collagen matrix deposition at the healing site was greatly improved after wound healing. We reviewed the relevant literature and found that the dual‐sided and time‐dependent effects of IL‐10 on wound healing has been described in previous studies. IL‐10 primarily functions by inhibiting the activity of various inflammatory cells, thereby mitigating the extent of the inflammatory response.^58^ Meanwhile, IL‐10 promotes the proliferation and migration of fibroblasts and epithelial cells, as well as promotes the formation of neovascularization, thereby accelerating the wound‐healing process.^59^ Another study has shown that topical addition of anti‐IL‐10 signaling after surgical debridement promoted the conversion of diabetic chronic wounds into acute fresh wounds, thereby facilitating healing.^60^ However, it was also shown that IL‐10 deficient mice have more myofibroblast differentiation and macrophage infiltration, thereby accelerating wound healing.^61^ Given the crucial role of the early inflammatory phase in initiating the wound‐healing response, clearing cellular debris, combating infection, and facilitating angiogenesis, it is important to consider the potential risk of converting to chronic wounds when applying IL‐10 early in the wound‐healing process.^62^ Meanwhile, clearance of myofibroblasts in the proliferation stage may also cause delayed wound healing. Although our study demonstrated the effectiveness of modIL‐10‐loaded ADSCs in preventing scar formation, more data are needed to adjust the concentration and timing of IL‐10.

Our study has certain limitations that should be acknowledged. First, our animal experiments employed rats of the same sex, which may introduce gender‐related bias in the results, potentially limiting the generalizability of our findings. Second, our experiments were confined to pathologic primary KFs. Further investigations are necessary to assess the safety of our platform by examining its effects on normal cells. In addition, more comprehensive controlled experiments are required to conclusively establish the superiority of our therapeutic platform over other mRNA delivery methods. To enhance their availability, future investigations may focus on exploring the utility of cell‐free products, such as exosomes, as therapeutic vectors, or the exploration of allogeneic cell therapy.

## 5 CONCLUSION

**FIGURE 8:**
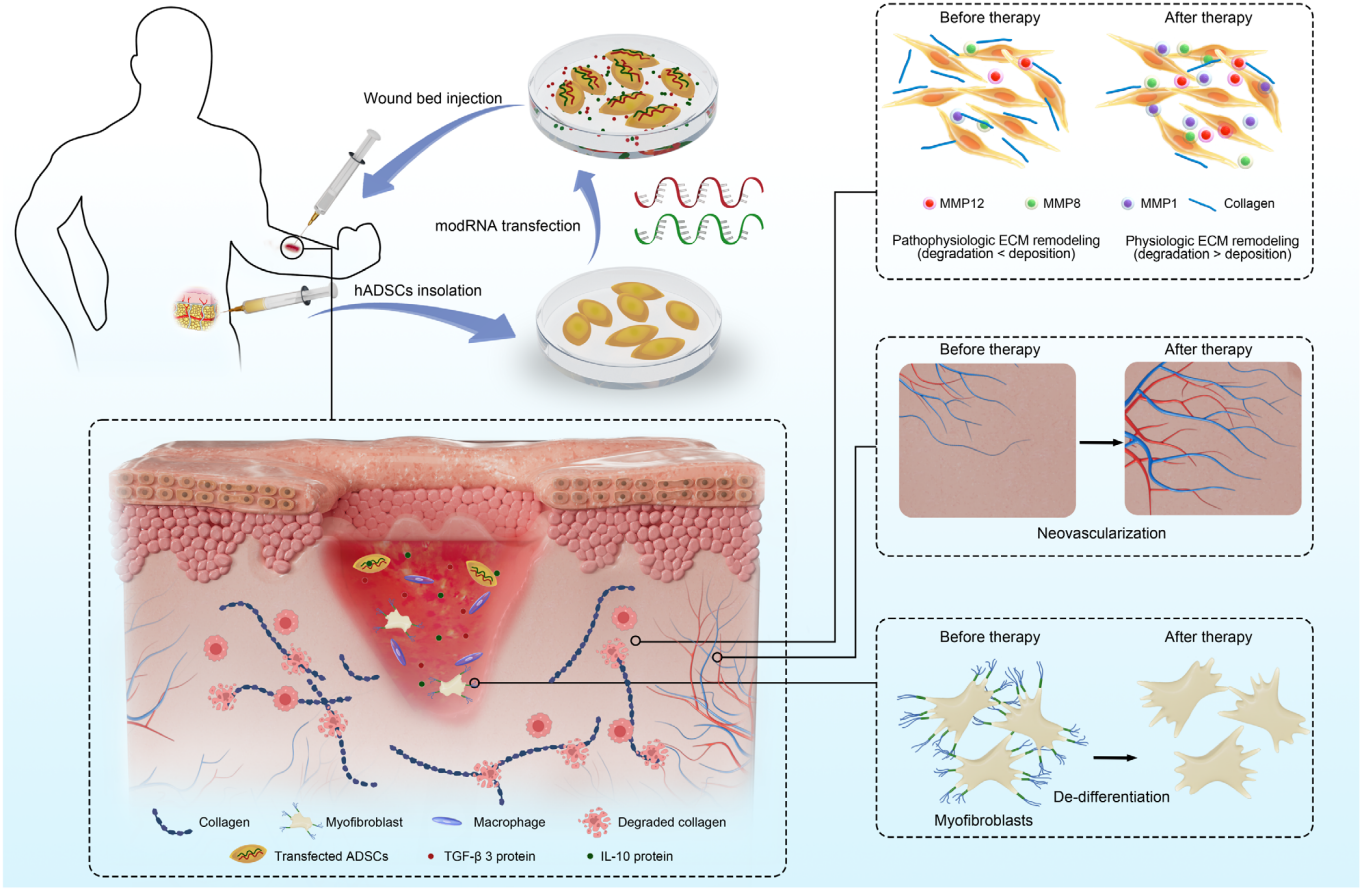
Schematic diagram depicting intradermal delivery of adipose‐derived stem cells (ADSCs) conditioned with therapeutic transforming growth factor‐β3 (TGF‐β3) and interleukin‐10 (IL‐10) messenger RNA as a novel therapeutic for the wound management of scar‐susceptible people.

We reported for the first time the application of modRNA technology in promoting scar‐less wound healing and demonstrated the multidimensional therapeutic potential of ADSCs overexpressing TGF‐β3 and IL‐10 in several preclinical models (Figure 8). The results represent a valuable step toward the mRNA drug‐based precision therapy for wound management, specifically tailored for individuals predisposed to scar formation.

## AUTHOR CONTRIBUTIONS

**Wei Wang:** Conceptualization (lead); data curation (equal); formal analysis (equal); investigation (equal); methodology (equal); visualization (lead); writing – original draft (lead); writing – review and editing (supporting). **Liang Chen:** Conceptualization (supporting); data curation (equal); formal analysis (equal); investigation (equal); resources (lead); writing – original draft (supporting). **Yuxin Zhang:** Data curation (equal); formal analysis (equal); investigation (equal); methodology (equal); visualization (supporting); writing – review and editing (supporting). **Heng Wang:** Conceptualization (supporting); investigation (supporting); methodology (supporting); visualization (supporting). **Dong Dong:** Conceptualization (supporting); data curation (supporting); formal analysis (supporting). **Jingjing Zhu:** Funding acquisition (supporting); methodology (supporting); supervision (supporting). **Wei Fu:** Conceptualization (supporting); project administration (lead); resources (supporting); supervision (supporting); writing – review and editing (lead). **Tianyi Liu:** Funding acquisition (lead); project administration (equal); supervision (lead).

## CONFLICT OF INTEREST STATEMENT

The authors declare no conflict of interest.

### PEER REVIEW

The peer review history for this article is available at https://www.webofscience.com/api/gateway/wos/peer-review/10.1002/btm2.10620.

## Supporting information


**DATA S1.** Supporting Information.

## Data Availability

The datasets used and/or analyzed during the current study are available from the corresponding author on a reasonable request.
