# Supplementary material for: Adipose‐derived stem cells enriched with therapeutic mRNA TGF‐β3 and IL‐10 synergistically promote scar‐less wound healing in preclinical models
Source: Bioeng Transl Med. 2023 Nov 10;9(2):e10620. doi: 10.1002/btm2.10620 (PMC10905533; doi:10.1002/btm2.10620)
Supplement: Supplementary file 1 — DATA S1. Supporting Information. [file BTM2-9-e10620-s001.docx]

**Supplementary Table 1.** Coding sequence for construction of human TGF-β3 and IL-10 modRNA

| **Human TGF-β3 CDS** | ATGAAGATGCACTTGCAAAGGGCTCTGGTGGTCCTGGCCCTGCTGAACTTTGCCACGGTCAGCCTCTCTCTGTCCACTTGCACCACCTTGGACTTCGGCCACATCAAGAAGAAGAGGGTGGAAGCCATTAGGGGACAGATCTTGAGCAAGCTCAGGCTCACCAGCCCCCCTGAGCCAACGGTGATGACCCACGTCCCCTATCAGGTCCTGGCCCTTTACAACAGCACCCGGGAGCTGCTGGAGGAGATGCATGGGGAGAGGGAGGAAGGCTGCACCCAGGAAAACACCGAGTCGGAATACTATGCCAAAGAAATCCATAAATTCGACATGATCCAGGGGCTGGCGGAGCACAACGAACTGGCTGTCTGCCCTAAAGGAATTACCTCCAAGGTTTTCCGCTTCAATGTGTCCTCAGTGGAGAAAAATAGAACCAACCTATTCCGAGCAGAATTCCGGGTCTTGCGGGTGCCCAACCCCAGCTCTAAGCGGAATGAGCAGAGGATCGAGCTCTTCCAGATCCTTCGGCCAGATGAGCACATTGCCAAACAGCGCTATATCGGTGGCAAGAATCTGCCCACACGGGGCACTGCCGAGTGGCTGTCCTTTGATGTCACTGACACTGTGCGTGAGTGGCTGTTGAGAAGAGAGTCCAACTTAGGTCTAGAAATCAGCATTCACTGTCCATGTCACACCTTTCAGCCCAATGGAGATATCCTGGAAAACATTCACGAGGTGATGGAAATCAAATTCAAAGGCGTGGACAATGAGGATGACCATGGCCGTGGAGATCTGGGGCGCCTCAAGAAGCAGAAGGATCACCACAACCCTCATCTAATCCTCATGATGATTCCCCCACACCGGCTCGACAACCCGGGCCAGGGGGGTCAGAGGAAGAAGCGGGCTTTGGACACCAATTACTGCTTCCGCAACTTGGAGGAGAACTGCTGTGTGCGCCCCCTCTACATTGACTTCCGACAGGATCTGGGCTGGAAGTGGGTCCATGAACCTAAGGGCTACTATGCCAACTTCTGCTCAGGCCCTTGCCCATACCTCCGCAGTGCAGACACAACCCACAGCACGGTGCTGGGACTGTACAACACTCTGAACCCTGAAGCATCTGCCTCGCCTTGCTGCGTGCCCCAGGACCTGGAGCCCCTGACCATCCTGTACTATGTTGGGAGGACCCCCAAAGTGGAGCAGCTCTCCAACATGGTGGTGAAGTCTTGTAAATGTAGCTGA |
| --- | --- |
| **Human IL-10 CDS** | ATGCACAGCTCAGCACTGCTCTGTTGCCTGGTCCTCCTGACTGGGGTGAGGGCCAGCCCAGGCCAGGGCACCCAGTCTGAGAACAGCTGCACCCACTTCCCAGGCAACCTGCCTAACATGCTTCGAGATCTCCGAGATGCCTTCAGCAGAGTGAAGACTTTCTTTCAAATGAAGGATCAGCTGGACAACTTGTTGTTAAAGGAGTCCTTGCTGGAGGACTTTAAGGGTTACCTGGGTTGCCAAGCCTTGTCTGAGATGATCCAGTTTTACCTGGAGGAGGTGATGCCCCAAGCTGAGAACCAAGACCCAGACATCAAGGCGCATGTGAACTCCCTGGGGGAGAACCTGAAGACCCTCAGGCTGAGGCTACGGCGCTGTCATCGATTTCTTCCCTGTGAAAACAAGAGCAAGGCCGTGGAGCAGGTGAAGAATGCCTTTAATAAGCTCCAAGAGAAAGGCATCTACAAAGCCATGAGTGAGTTTGACATCTTCATCAACTACATAGAAGCCTACATGACAATGAAGATACGAAACTGA |

**Supplementary Table 2.** Human primers used in qRT-PCR analysis.

| **Primers** |  |
| --- | --- |
| **FN1 forward** | GAGAATAAGCTGTACCATCGCAA |
| **FN1 reverse** | CGACCACATAGGAAGTCCCAG |
| **MMP1 forward** | GGGCTGAAAGTGACTGGGAAACC |
| **MMP1 reverse** | TGCTTGACCCTCAGAGACCTTGG |
| **MMP8 forward** | AAGTGGGAACGCACTAACTTG |
| **MMP8 reverse** | GGATTCCATTGGGTCCATCAAAT |
| **MMP12 forward** | TCCTGATGTGGGTGAATACAATG |
| **MMP12 reverse** | GCCATCGTGAAGTCTGGTAAAAT |
| **COL1A1 forward** | GTGCGATGACGTGATCTGTGA |
| **COL1A1 reverse** | CGGTGGTTTCTTGGTCGGT |
| **COL3A1 forward** | GCTACGGCAATCCTGAACTTCCTG |
| **COL3A1 reverse** | GCAACCATCCTCCAGAACTGTGTAG |
| **TGF-β1 forward** | ACTTGCACCACCTTGGACTTC |
| **TGF-β1 reverse** | GGTCATCACCGTTGGCTCA |
| **ACTA2 forward** | GTGTTGCCCCTGAAGAGCAT |
| **ACTA2 reverse** | GCTGGGACATTGAAAGTCTCA |
| **TGF-β3 forward** | TTCCGCTTCAATGTGTCCTCAGTG |
| **TGF-β3 reverse** | CGATCCTCTGCTCATTCCGCTTAG |
| **IL-10 forward** | ACTGCTCTGTTGCCTGGTCCTC |
| **­IL-10 reverse** | GCCTTGATGTCTGGGTCTTGGTTC |
| **GAPDH forward** | GGGAGCCAAAAGGGTCATCATCTC |
| **GAPDH reverse** | GAGGGGCCATCCACAGTCTTC |

**Supplementary Figure 1**


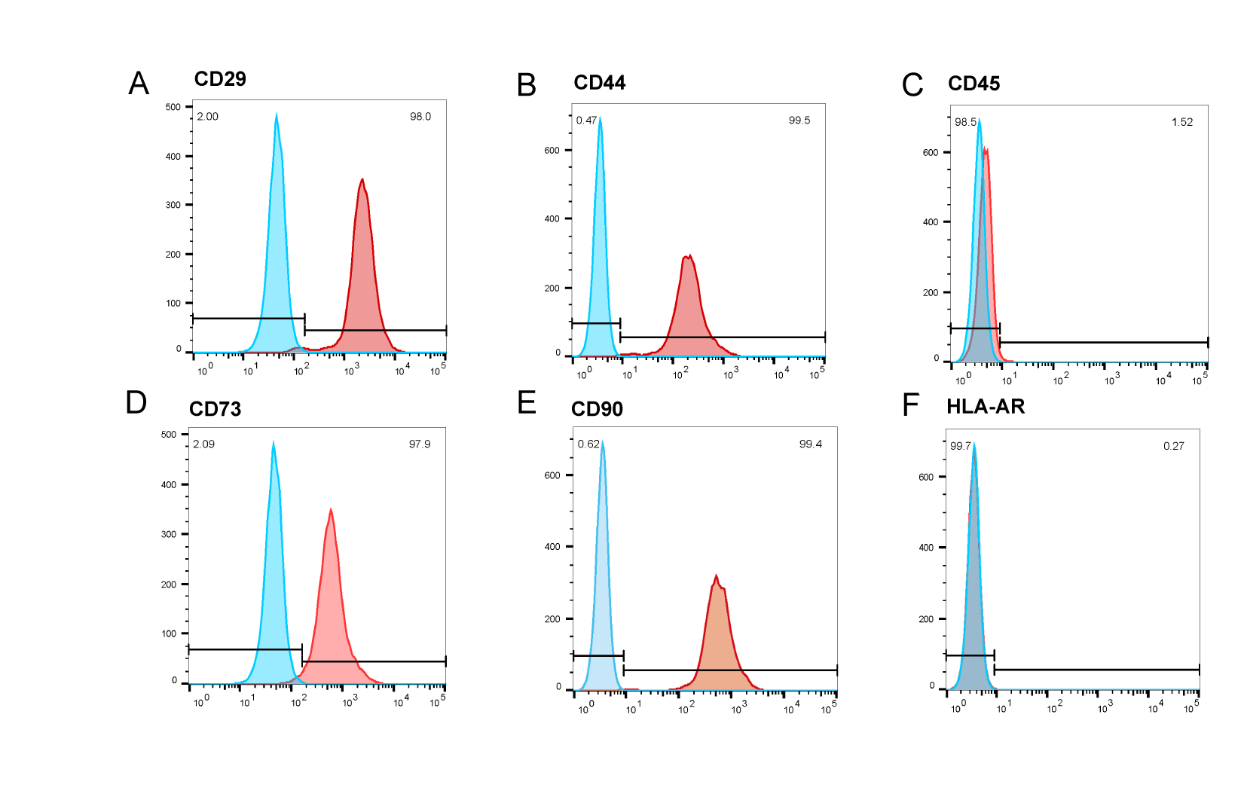


**Figure S1. A-F.** Flow cytometry analysis of surface markers in the ADSCs, including CD29 (A), CD44 (B), CD45 (C), CD73 (D), CD90 (E) and HLA-AR (F). The blue peaks represent blank control and the red peaks represent marked ADSCs.

**Supplementary Figure 2**


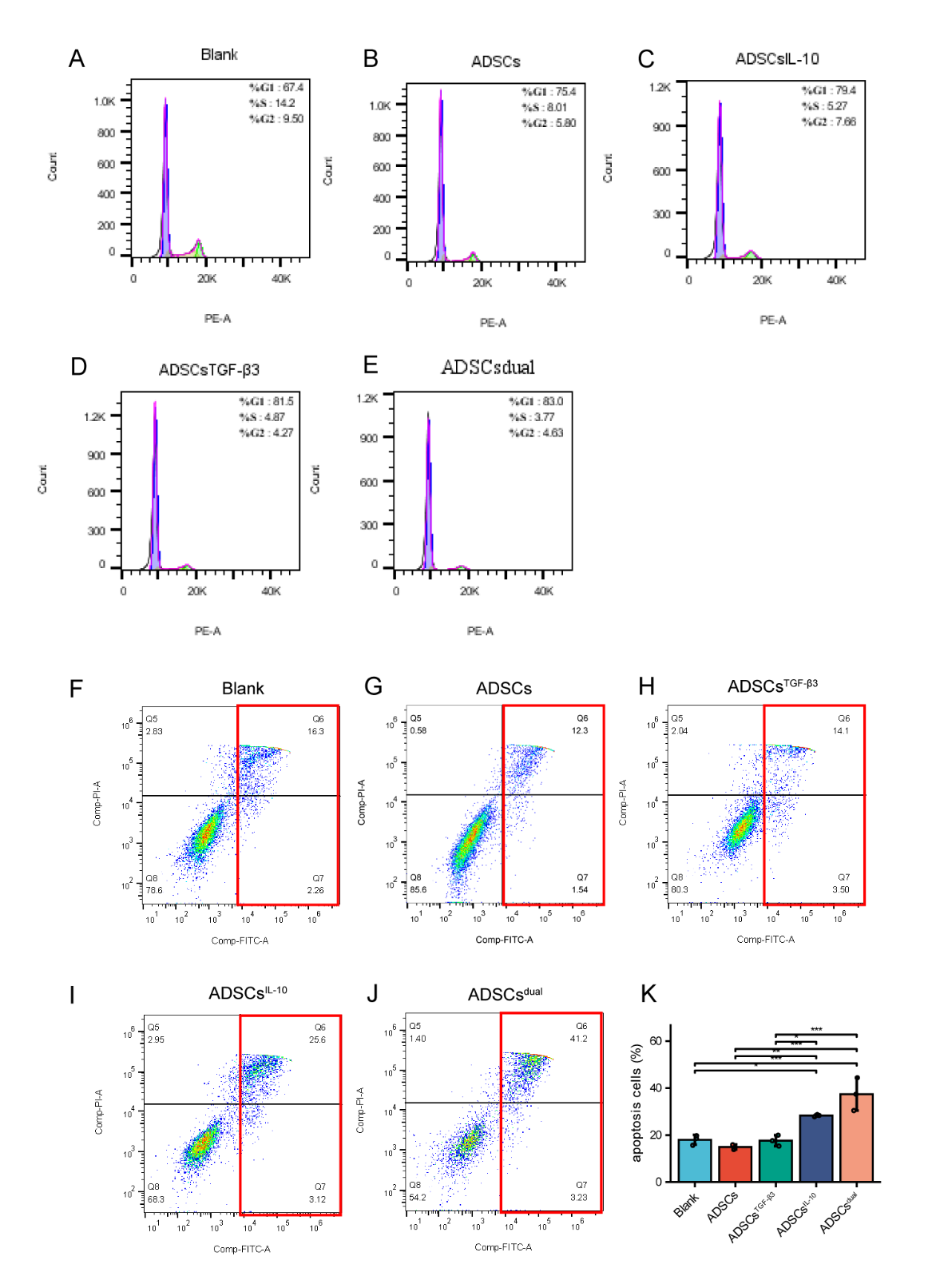


**Figure S2.** **A-E.** Representative histograms showing the cell cycle distrubution of KFs co-cultured with ADSCs (B), ADSCs^TGF-β3^ (C), ADSCs^IL-10^ (D) and ADSCs^dual^ (E) for 24 h, primary KFs (A) were used as the control. **F-J.** KFs were co-cultured with ADSCs (G), ADSCs^TGF-β3^ (H), ADSCs^IL-10^ (I) and ADSCs^dual^ (J) for 48 h, and cell apoptosis was detected by flow cytometry after annexin V and propidium (PI) dual-labeling, and representative images of the apoptotic cells were shown (n = 3 samples). Primary KFs (F) were used as the control. **K.** Bar chart showing the apopotosis rates of KFs in different groups. Values were presented as mean ± standard deviation (SD), with n = 3 samples per group. Statistical significance was determined using *p < 0.05, **p < 0.01, ***p < 0.001.

**Supplementary Figure 3**


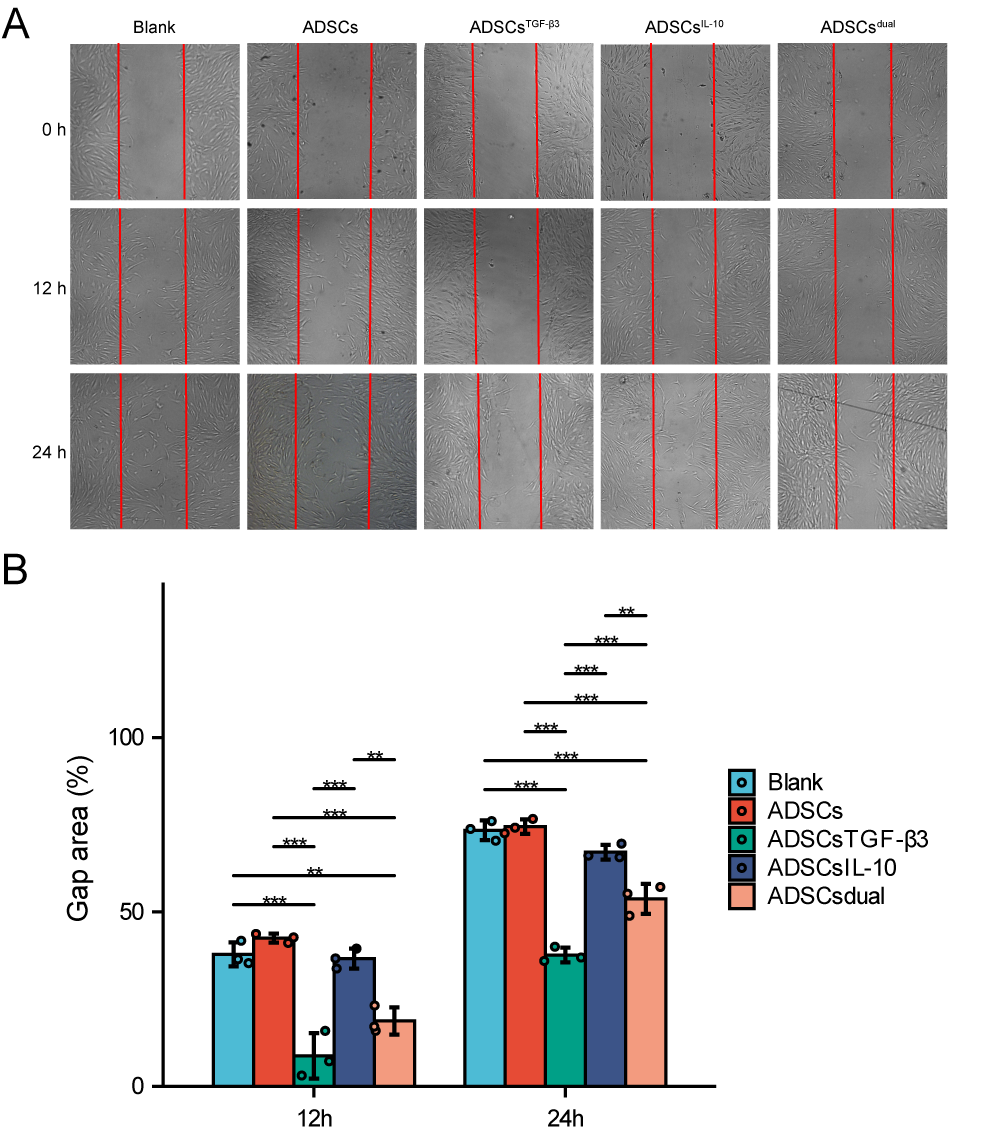


**Figure S3. A.** Scratch assay evaluating the migration ability of KFs treated with ADSCs, ADSCs^TGF-β3^, ADSCs^IL-10^, or ADSCs^dual^. Representative images of gap areas at 0, 12, and 24 hours were shown. Untreated primary KFs were used as the control. **B.** Comparison of gap areas of KFs after 12 or 24 hours treatment.

**Supplementary Figure 4**


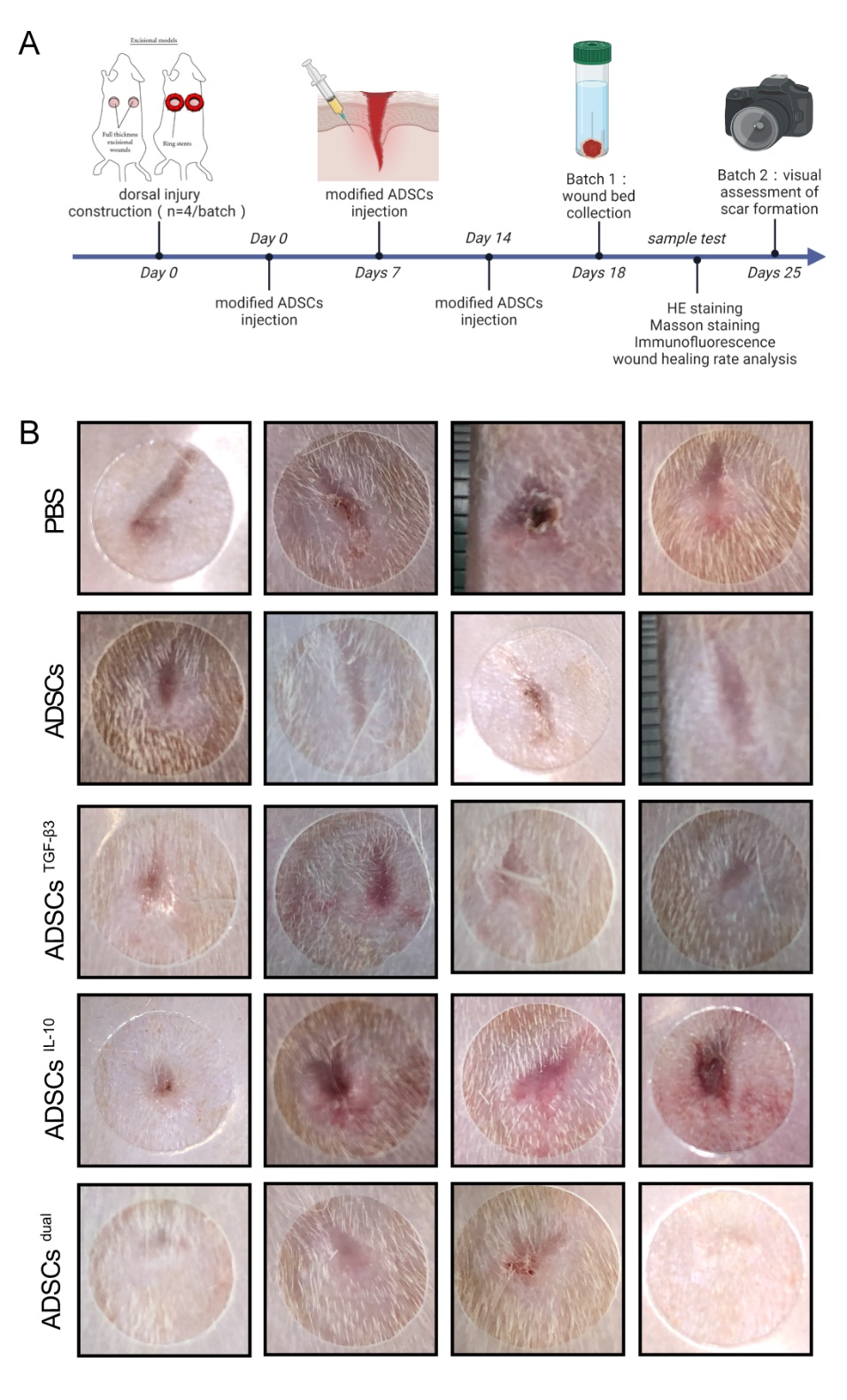


**Figure S4. A.** Schematic of the animal experimental approach: Two 1-cm excisional wounds were created on the back of each rat. A silicone splinting ring was tightly adhered and secured (with four interrupted sutures of 6.0 nylon) to the skin around the wound to prevent wound closure caused by skin contraction. On days 0, 7, and 14 after modeling, 0.7 × 10^6^ modRNAs-enriched hADSCs in 100 μl of PBS were injected into the dermis. Meanwhile, we also topically applied 0.3 × 10^6^ cells in 20 μl of growth factor–reduced Matrigel (BD Biosciences) onto the wound bed. One of the wounds per animal (n=4) was photographed on days 0, 5, 10, 15, and 25 to observe wound healing and scar formation. The other wounds per animal(n=4) was sampled on day 18 for histological analysis. **B.** Representative photographs of scar formation in the different groups of rats on day 25.

**Supplementary Figure 5**


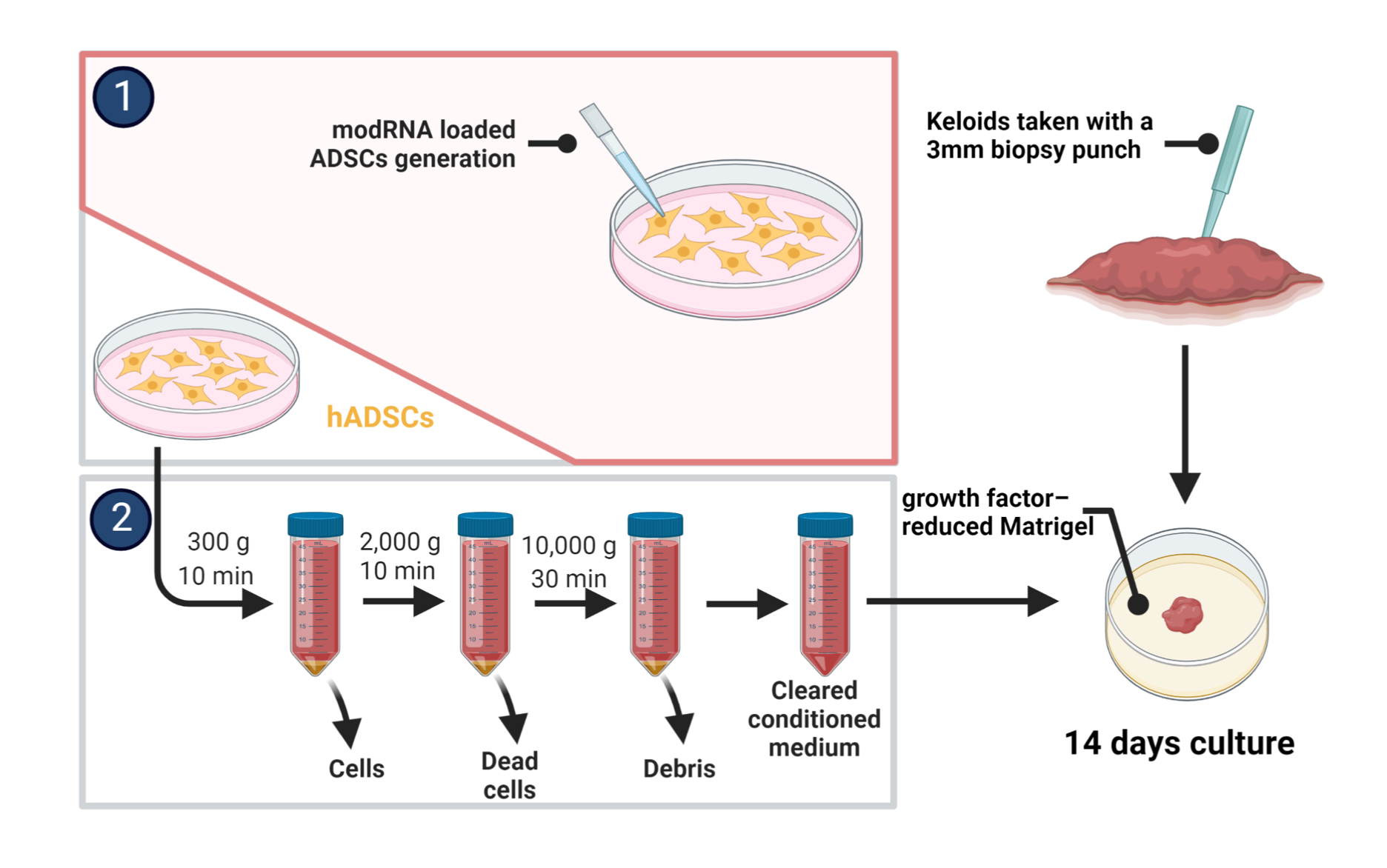


**Figure S5.** Schematic of the ex vivo keloid culture.

**Supplementary Figure 6**


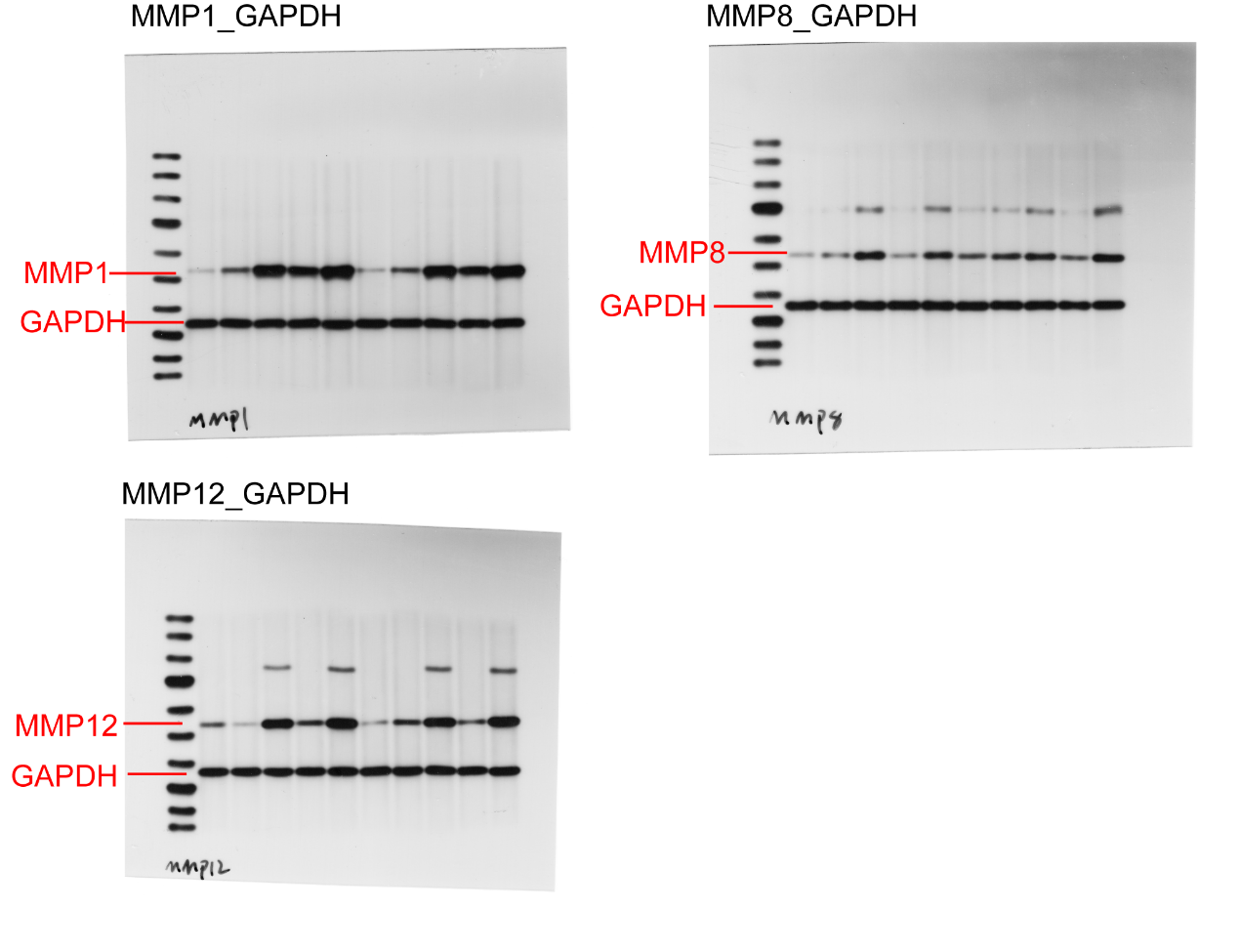


**Figure S6.** The full blots for the blots in Figure 3F.

lane 1: Untreated_1

lane 2: ADSCs_1

lane 3: ADSCs^TGF-β3^_1

lane 4: ADSCs^IL-10^_1

lane 5: ADSCs^dual^_1

lane 6: Untreated_2

lane 7: ADSCs_2

lane 8: ADSCs^TGF-β3^_2

lane 9: ADSCs^IL-10^_2

lane 10: ADSCs^dual^_2

**Supplementary Figure 7**


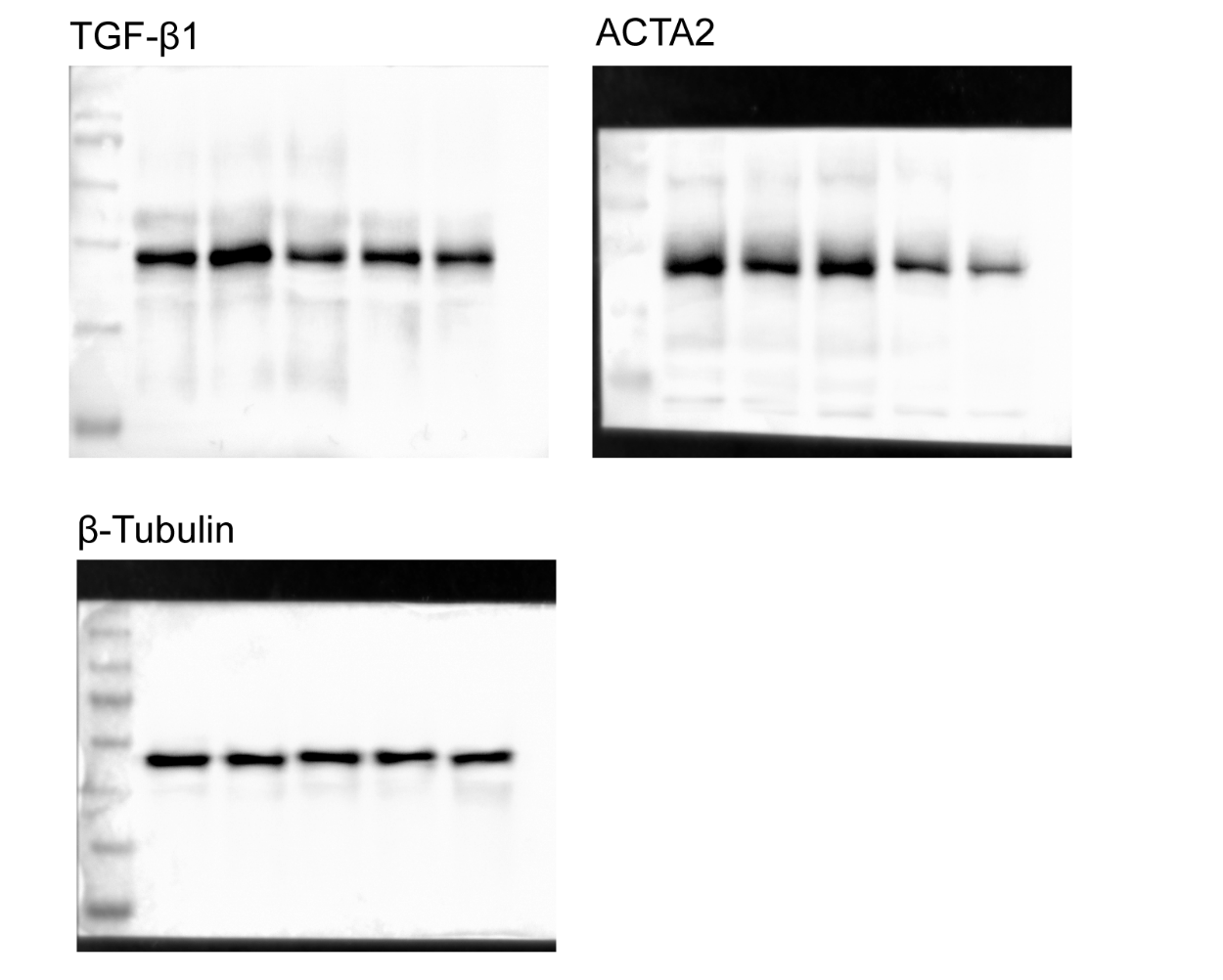


**Figure S7.** The full blots for the blots in Figure 4F.

lane 1: Untreated_1

lane 2: ADSCs_1

lane 3: ADSCs^TGF-β3^_1

lane 4: ADSCs^IL-10^_1

lane 5: ADSCs^dual^_1

**Supplementary Figure 8**


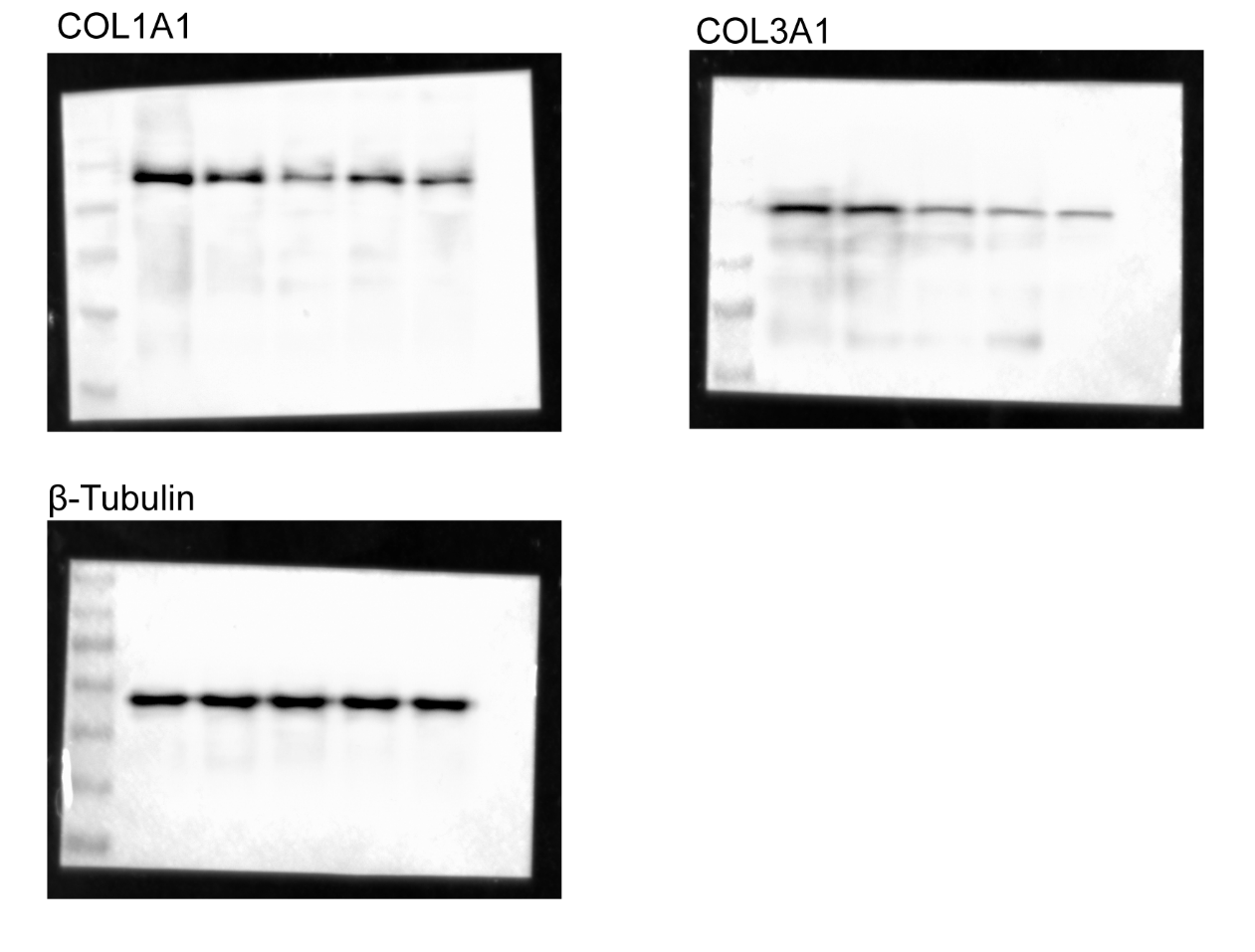


**Figure S8.** The full blots for the blots in Figure 7F.

lane 1: Untreated_1

lane 2: ADSCs_1

lane 3: ADSCs^TGF-β3^_1

lane 4: ADSCs^IL-10^_1

lane 5: ADSCs^dual^_1
